# Supplementary material for: Prevention of aspartimide formation during peptide synthesis using cyanosulfurylides as carboxylic acid-protecting groups
Source: Nat Commun. 2020 Feb 20;11:982. doi: 10.1038/s41467-020-14755-6 (PMC7033154; doi:10.1038/s41467-020-14755-6)
Supplement: Supplementary file 1 — Supplementary Information [file 41467_2020_14755_MOESM1_ESM.pdf]

Supplementary Information

Prevention of aspartimide formation during peptide synthesis using cyanosulfurylides as carboxylic acid protecting groups

Kevin Neumann et al.

Table of Content

1. **Supplementary Figures…..………………...…………………………….……..…….3**

2. **Supplementary Tables…………………………………………………………10**

3. **Supplementary Methods…………………………………………..……......11**

4. **Supplementary References………………..………………………………......41**

1. Supplementary Figures

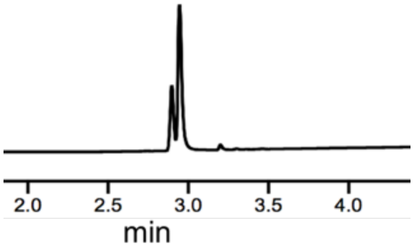

Supplementary Figure 1. **Stability in presence of TEMPO.** LC-MS trace of N-Fmoc-Asp(CSY)-OH **5** (t_r = 2.90 min) incubated with TEMPO (t_r = 2.95 min).

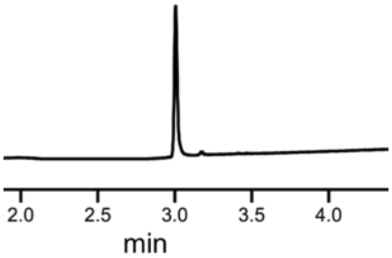

Supplementary Figure 2. **Stability in presence of H_2O_2.** LC-MS trace of N-Fmoc-Asp(CSY)-OH **5** (t_r = 3.02 min) incubated with H_2O_2.

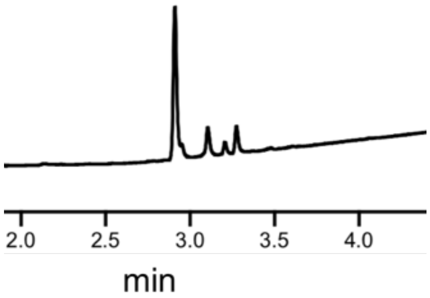

Supplementary Figure 3. **Stability in presence of TEMPO and $NaNO_2$.** LC-MS trace of N-Fmoc-Asp(CSY)-OH **5** (t_r = 2.90 min) incubated with TEMPO and $NaNO_2$ in AcOH.

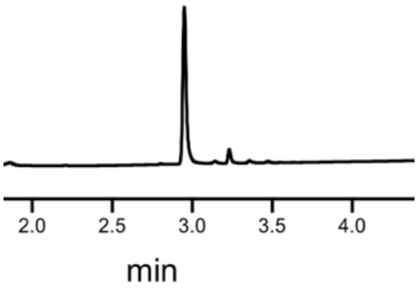

Supplementary Figure 4. **Stability in presence of an Bromoacetontrile.** LC-MS trace of N-Fmoc-Asp(CSY)-OH **5** (t_r = 2.90 min) incubated with Bromoacetonitrile.

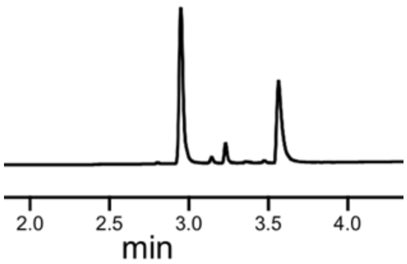

Supplementary Figure 5. **Stability in presence of (Diacetoxyiodo)benzene.** LC-MS trace of N-Fmoc-Asp(CSY)-OH **5** (t_r = 2.90 min) incubated with (Diacetoxyiodo)benzene (t_r = 3.10 min).

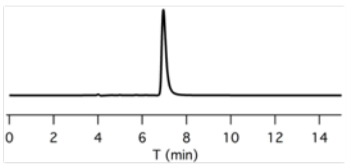

Commercially available reference substances for comparison:

Fmoc-D-Asp(OH)-O^{t}Bu:

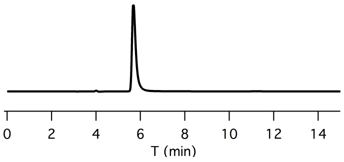

Fmoc-L-Asp(OH)-O^{t}Bu:

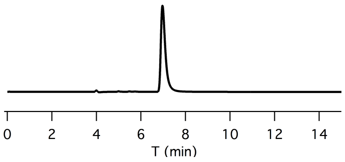

Fmoc-D-Asp(OH)-O^{t}Bu and Fmoc-L-Asp(OH)-O^{t}Bu mixture 1:1:

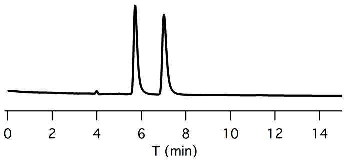

Sample with added standard of 1:1 mixture:

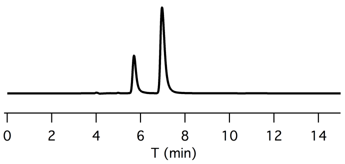

Supplementary Figure 6. **Deprotection of CSY on larger scale.** Chiral normal-phase HPLC of deprotected Fmoc-Asp(OH)-O^{t}Bu **4** determining the stereo configuration after removal of CSY.

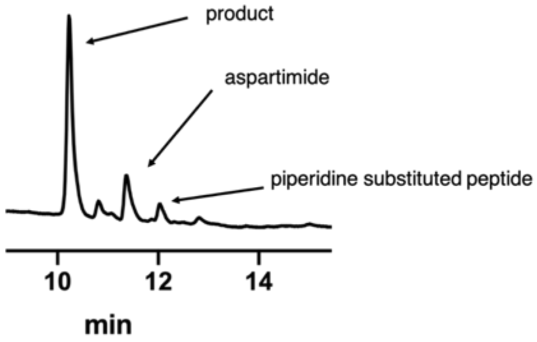

Supplementary Figure 7. **Comparison to Asp(OMpe).** Besides model peptides **6a** and **6b**, peptide **S1** was synthesized using commercially available Fmoc-Asp(OMpe) bearing a sterically hindered ester. The peptide was incubated in the same way in 20% piperidine in DMF as model peptides **6a** and **6b**. The resin was then treated with TFA/DODT/H_2O (95:2.5:2.5, v/v) for 2 h. HPLC analysis revealed less formed aspartimide compared to model peptide **6a**. Yet, a significant amount of aspartimide and piperidine substituted peptide was observed. In contrast, peptide **6b** bearing Asp(CSY) does not display any aspartimide (Figure 2F).

A)

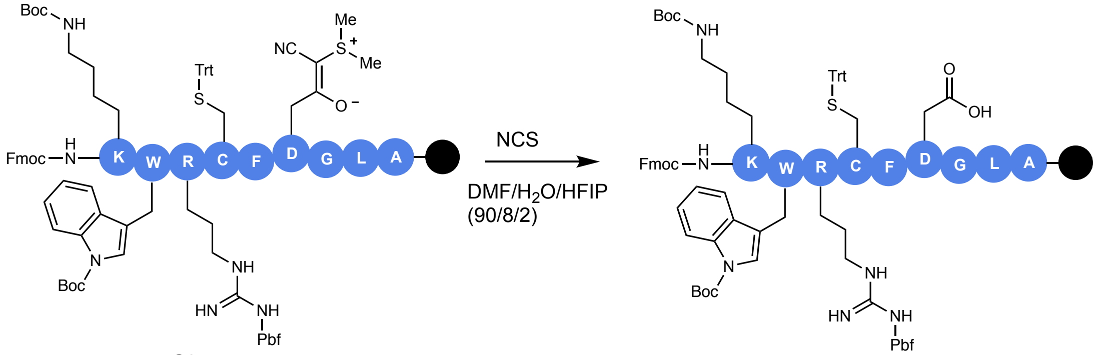

S2

B)

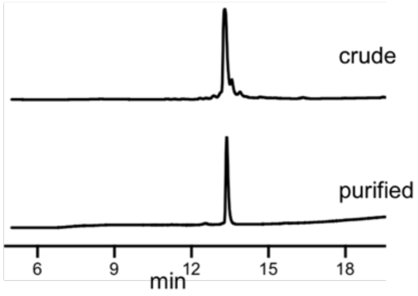

Supplementary Figure 8. **On-resin deprotection of the CSY**. A) Deprotection of **S2** was carried out by treating Rink-amide resin with NCS (from 1.5 equiv) in DMF/H_2O/HFIP (90:8:2, v:v:v) for 2 min. B) A small amount of aspartimide is observed in the crude HPLC.

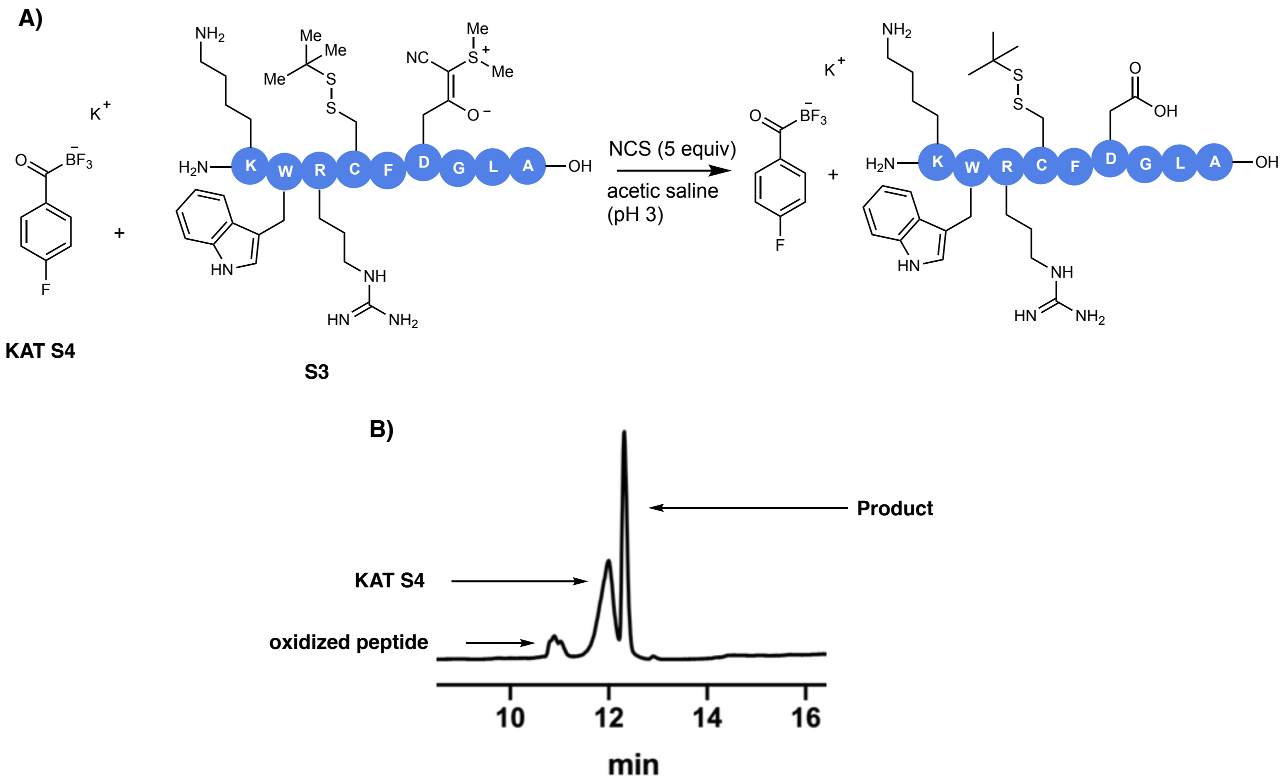

Supplementary Figure 9. **Detection of N-Chloroamines**. Potassium acyl trifluoroborates undergo rapid ligation with N-chloroamines at low concentration resulting in stable amide linkages. To determine if any N-chloroamines form in the course of the reaction, peptide **S3** (1 equiv) was incubated with an excess of NCS (5 equiv) and KAT **S4** (15 equiv) in acidic saline (pH 3). Reaction was followed by HPLC. CSY deprotection of peptide **S3** was observed within minutes; as the only minor byproduct we observed disulfide cleavage and oxidized cysteine moieties, which we attributed to the large excess of NCS. We did not observe any traces of the expected ligation product of peptide and KAT. **A)** Incubation of peptide **S3** with NCS (5 equiv) in the presence of an excess of KAT S4 (15 equiv). **B)** HPLC traces obtained after 10 min show only CSY deprotected peptide and a minor amount of oxidized peptide due to the large excess of NCS.

A)

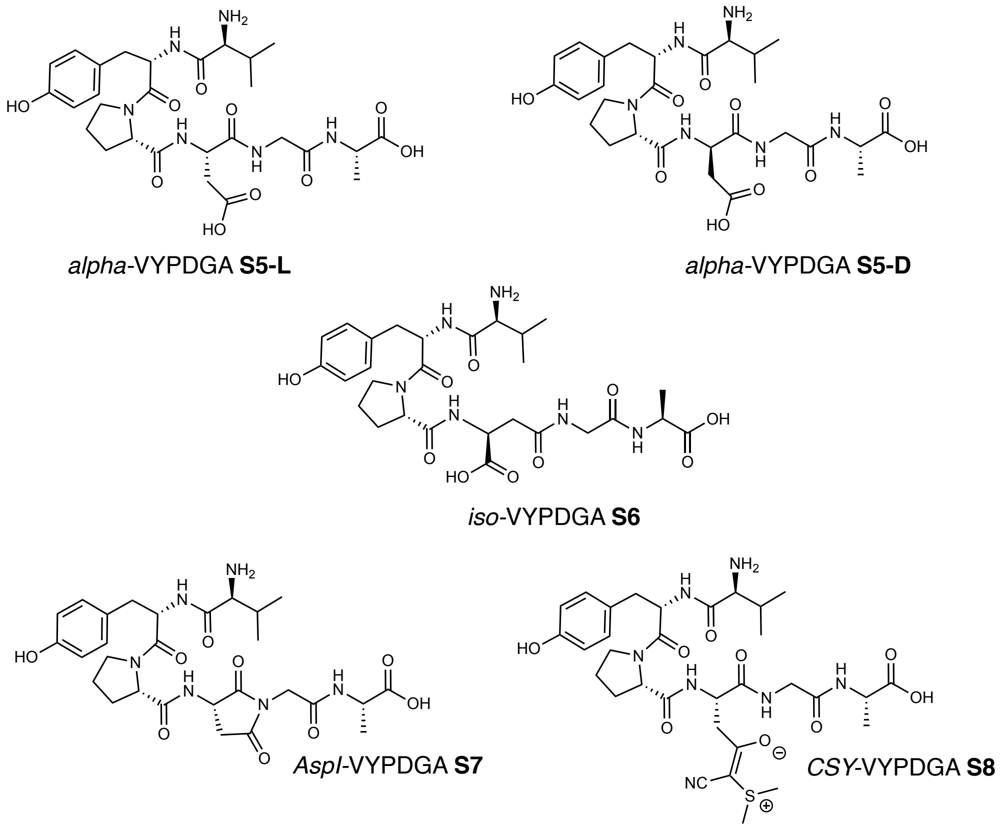

B)

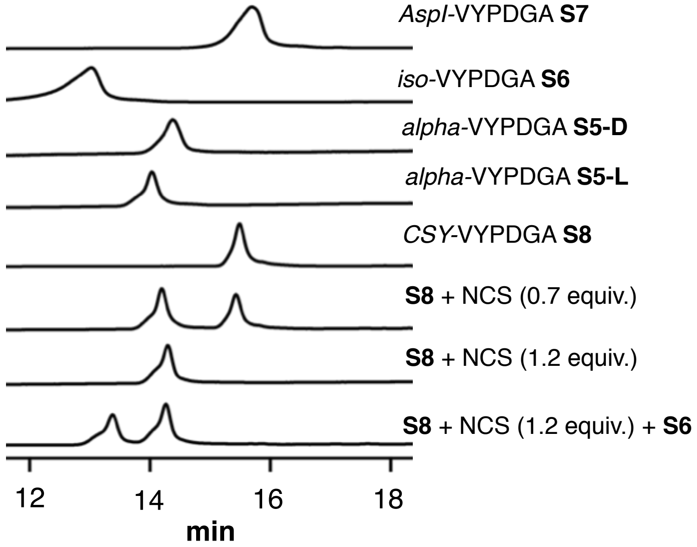

Supplementary Figure 10. **Isomerization studies during CSY removal. A)** Structures of synthesized model peptides including *alpha*-L-Asp-, *alpha*-D-Asp-, *beta*-, aspartimide- and CSY containing derivatives. **B)** HPLC traces purified peptides **S5** to **S8** and monitoring of removal of CSY. In addition, the fully deprotected peptides was spiked with the *beta*-peptide **S6** reference.

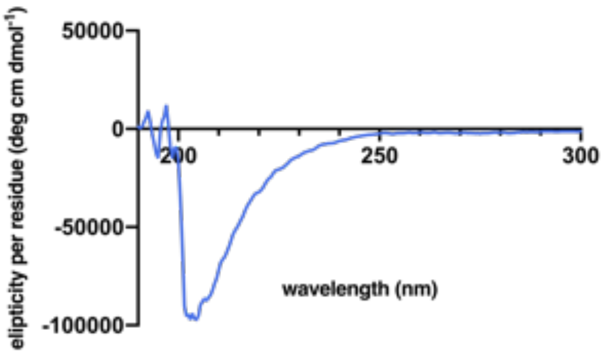

Supplementary Figure 11. **CD spectrum of folded LDLa 11.** Folding was carried out according to literature.[1] Briefly, the peptide was dissolved (150 µg mL^{-1}) in refolding buffer at pH 8.5 containing of GSH (3 mM), GSSG (0.3 mM), Tris-HCl (50 mM), NaCl (150 mM) and $CaCl_2$ (2.5 mM) at 4 °C. We observed the best results by pre-cooling the folding buffer.

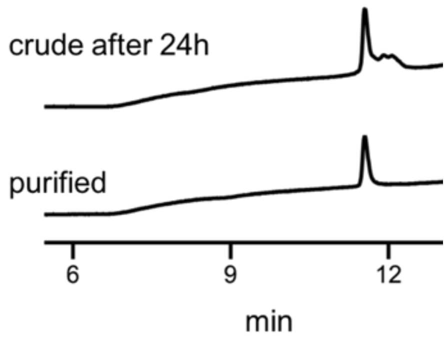

Supplementary Figure 12. **HPLC traces of LDLa 11.** Crude HPLC trace of LDLa 11 after 24h in folding buffer and preparative HPLC purified LDLa.

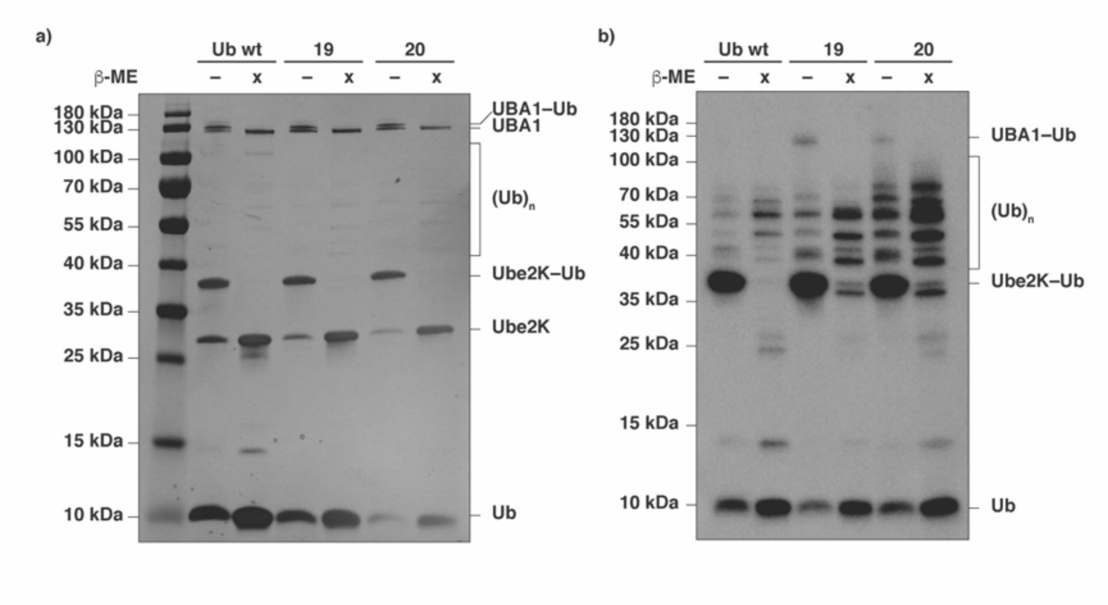

Supplementary Figure 13. **Ubiquitination Assays.** Poly-ubiquitination of **19** and **20** compared to recombinant ubiquitin. Ubiquitins were incubated with UBA1, Ube2K and ATP and loaded onto a SDS-page gel under reducing or non-reducing conditions. Poly-ubiquitin chains are observed for all ubiquitin variants. **a)** Silver stain of poly-ubiquitin chain formation. Under non-reducing conditions the formation of a thiol-sensitive band is observed, corresponding to the Ub-Ube2K thioester. **b)** Western blot against ubiquitin of poly-ubiquitin chain formation. The formation of high molecular weight poly-ubiquitin chains is observed.

2. Supplementary Table

Supplementary Table 1. Reagents that were used to determine stability of the CSY in presence of oxidizing reagents, radicals and electrophiles.

Entry	Reagent	Solvent
1	TEMPO (10 equiv)	CH_3CN
2	H_2O_2 (20 equiv)	CH_3CN/H_2O (1:1, v:v)
3	TEMPO/$NaNO_2$ (10 equiv)	AcOH
4	$BrCH_2CN$ (15 equiv)	CH_3CN
5	(Diacetoxyiodo)benzene (10 equiv)	CH_3CN

3. Supplementary Methods

Reagents

Fmoc-amino acids with suitable side-chain protecting groups, HCTU (*O*-(1*H*-6-chlorobenzotriazol-1-yl)- *N*,*N*,*N*',*N*'-tetramethyluroniumhexafluorophosphate) and HATU (1-[bis(dimethylamino)methylene]-1H-1,2,3-triazolo[4,5-b]pyridinium 3-oxide hexafluorophosphate) were purchased from Peptides International (Louisville, KY, USA) and ChemImpex (Wood Dale, IL, USA). Recombinant ubiquitin was obtained from Sigma-Aldrich. Recombinant UBA1 and Ube2K were purchased from Enzo Life Sciences. Antibodies were purchased from Cell-Signaling (anti-ubiquitin produced in rabbit, HRP-linked-anti-rabbit antibody produced in goat). Solvents for flash column chromatography (EtOAc, MeOH, CH_2Cl_2) were of technical grade and distilled prior to use. HPLC grade CH_3CN from Sigma-Aldrich was used for analytical and preparative HPLC purification. DMF (> 99.8%) from Sigma-Aldrich was directly used without further purification for solid phase peptide synthesis. Other commercially available reagents and solvents were purchased from Sigma-Aldrich (Buchs, Switzerland), Acros Organics (Geel, Belgium) and TCI Europe (Zwijndrecht, Belgium).

Characterization

^{1}H and ^{13}C NMR spectra were recorded on Bruker AVIII400 or Bruker AVIII500 spectrometers. Chemical shifts for ^{1}H NMR (400, and 500 MHz) and ^{13}C NMR (101 and 151 MHz) are expressed in parts per million and are referenced to residual undeuterated solvent signals. Coupling constants are reported in Hertz (Hz) and the corresponding splitting patterns are indicated as follows: s, singlet; bs, broad singlet; d, doublet; dd, doublet of doublet; ddd, doublet of doublet of doublet; td, triplet of doublet; t, triplet; m, multiplet; appt d, apparent triplet of a doublet; d apt, doublet of an apparent triplet; d appt d, doublet of an apparent triplet of a doublet. High-resolution mass spectra were recorded by the Molecular and Biomolecular Analysis Service (MoBiAS) at ETH Zurich, either with a Bruker maXis instrument (ESI-MS measurements) equipped with an ESI source and a Qq-TOF detector or with a Bruker solariX instrument (MALDI-FTICR-MS) using 4-hydroxy-α-cyanocinnamic acid as matrix. High-resolution mass spectra of large peptides and proteins were recorded by the functional genomics centre Zurich using a Synapt G2_Si mass spectrometer.

Circular dichroism (CD) analysis

CD spectra were recorded on a Jasco J715 spectropolarimeter in a 1 mm quartz cell at 20 ºC. Samples were measured in a potassium phosphate solution (25 mM, pH 7.0) at a concentration of 0.25 mg/mL. Spectra of three independent samples were measured, background subtracted, averaged, and plotted as their mean with ± S.D.

Reactions and purification

All reactions were performed using standard techniques under an atmosphere of N_2. Reactions and fractions from flash column chromatography were monitored by thin layer chromatography using aluminum TLC plates (Merck, TLC Silica gel 60 W F_{254}s) and visualized by staining with basic $KMnO_4$ solution or acidic ninhydrin solution. Flash column chromatography was performed on Sigma-Aldrich SiO_2 Type F60 (230-400 mesh) using a forced flow of air at 0.5-1.0 bar. Unless otherwise stated, peptides and protein segments were analyzed and purified by reversed phase high performance liquid chromatography (RP-HPLC) on Jasco analytical and preparative instruments equipped with dual pumps, mixer and in-line degasser, a variable wavelength UV detector (simultaneous monitoring of the eluent at 220 nm, 254 nm and 301 nm) and a Rheodyne injector fitted with a 20 or 1000 µL injection loop. If required, the columns were heated using an Alltech column heater or a H_2O bath (preparative HPLC). The mobile phase for RP-HPLC were Milipore-H_2O containing 0.1% (v/v) TFA and HPLC grade CH_3CN containing 0.1% (v/v) TFA. Analytical HPLC was performed on Shiseido Capcell Pak C18 MGII (5 µm, 4.6 mm I.D. x 250 mm) or Shiseido Capcell Pak C18 (UG 80, 5 µm, 4.6 mm I.D. x 250 mm) columns at a flow rate of 1 ml/min. Preparative HPLC was performed on Shiseido Capcell Pak MGII (5 µm, 20 mm I.D. x 250 mm) at a flow rate of 10 mL/min.

Peptide amounts below 10 mg were determined in solution by UV-absorption (λ = 280 nm) with extinction coefficient of given peptide.

General *analytical* HPLC methods:

- flow 1 mL/min, isocratic 10% CH_3CN for 3 min, then gradient from 10% to 90% CH_3CN in 22 min

General *analytical* LC-MS methods:

- flow 0.5 mL/min, isocratic 2% CH_3CN for 0.5 min, then gradient from 2% to 98% CH_3CN in 6 min

List of general *preparative* HPLC methods:

- Method A: flow 10 mL/min, isocratic 10% CH_3CN for 5 min, then gradient from 20% to 70%

CH_3CN in 28 min.

- Method B: flow 10 mL/min, isocratic 10% CH_3CN for 5 min, then gradient from 30% to 80% CH_3CN in 28 min.
- Method C: flow 40 mL/min, isocratic 20% CH_3CN for 10 min, then gradient 20% to 70% CH_3CN in 40min.
- Method D: flow 10 mL/min, isocratic 10% CH_3CN for 5 min, then gradient from 10% to 50% CH_3CN in 28 min.

Solid phase peptide synthesis

Loading of amino acids on solid support was performed as followed:

- chloro-trityl resin: the amino acid (1.20 equiv of desired loading) was dissolved in CH_2Cl_2 (500mM). DIPEA (2 equiv) was added to the solution. The solution was given to preswollen chloro-trityl resin and was shaken for 1h. The resin was washed three times with CH_2Cl_2. Remaining chloro-trityl moieties were capped with CH_2Cl_2/MeOH/DIPEA (17:2:1, v:v:v) for 1 min. The resin was washed with CH_2Cl_2 and used freshly for SPPS.
- Rink-amide resin: Rink amide resin was first Fmoc deprotected using 20% Piperidine in DMF (2 x 10 min). The resin was washed six times with DMF. The amino acid (1.20 equiv of aimed loading) and HCTU (1.18 equiv of aimed loading) was dissolved in DMF (400 mM). NMM (2.40 equiv) was added to the solution. The solution was given to preswollen Rink-amide resin and shaken for 6 h. The resin was washed three times with DMF and CH_2Cl_2. Remaining active amines were capped with DMF/DIPEA/acetic acid anhydride (10:2:1, v:v:v). The resin was washed with CH_2Cl_2 and stored at -20°C.

Peptides were synthesized on a Multisyntech Syro I parallel synthesizer using Fmoc-SPPS chemistry. The following Fmoc amino acids with side-chain protection groups were used: Fmoc-Ala-OH, Fmoc-Arg(Pbf)-OH, Fmoc-Asn(Trt)-OH, Fmoc-Asp(OtBu)-OH, Fmoc-Cys(S^tBu)-OH, Fmoc-Gln(Trt)-OH, Fmoc-Glu(OtBu)-OH, Fmoc-Gly-OH, Fmoc-His(1-Trt)-OH, Fmoc-Ile-OH, Fmoc-Leu-OH, Fmoc-Lys(Boc)-OH, Fmoc-Met-OH, Fmoc-Nle-OH, Fmoc-Phe-OH, Fmoc-Pro-OH, Fmoc-Ser(tBu)-OH, Fmoc-Thr(tBu)-OH, Fmoc-Trp(Boc)-OH, Fmoc-Tyr(tBu)-OH, Fmoc-Val-OH.

Western Blot

Samples were resolved using SDS-PAGE on a 8-16% polyacrylamide gel (Bio-Rad). Samples were electrophoretically transferred to a PVDF membrane (Bio-Rad) and blocked using 10 wt% skim milk in TBS-T for 1 h at room temperature. Membrane was washed using TBS-T and incubated with ubiquitin-antibody (Cell-Signaling, cat. No. 3933, 1:1,000) in TBS-T (1 wt% BSA) for 1h at room temperature. After washing, the membrane was incubated with HRP-linked-anti-rabbit antibody (Cell Signaling, cat. No 7074 1:5,000). The membrane was washed successively with TBS-T, TBS and Milipore-H_2O. Bands were visualized with a chemiluminescent reagent (Clarity Western ECL, Bio-Rad) on a Chemidoc MP imaging system.

Silver Staining

Samples were resolved using SDS-PAGE on a 8-16% polyacrylamide gel (Bio-Rad). Membrane was washed with 50 vol% AcOH in Milipore-H_2O followed by Milipore-H_2O. The gel was then incubated for 1 min in 0.02 wt% $Na_2S_2O_3$, and washed with Milipore-H_2O. The membrane was incubated with 0.1 wt% $AgNO_3$, 0.08 wt% formaldehyde (37%) for 20 min at 4 ºC, and washed with Milipore-H_2O. The bands were visualized using a 2 wt% Na_2CO_3, 0.04 wt% formaldehyde (37%).

(Cyanomethyl)-Dimethylsulfonium bromide

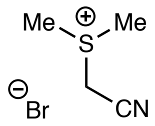

A mixture of bromoacetonitrile (7.15 mL, 103 mmol, 1.00 equiv) and dimethyl sulfide (7.53 mL, 103 mmol, 1.00 equiv) was stirred for 12 h. The resulting white solid was broken apart and washed with Et_2O. Residual solvent was removed under reduced pressure to give **(Cyanomethyl)-Dimethylsulfonium bromide** (16.8 g, 92.3 mmol, 90%)

The obtained spectral data were identical to those reported in literature.[2]

Cyanosulfurylide 1

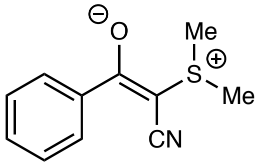

Benzoic acid **2** (2.0 g, 16.4 mmol, 1.00 equiv) was dissolved in CH_2Cl_2 (100 mL). *N*,*N*-Diisopropylethylamine (8.4 mL, 49.2 mmol, 3.00 equiv) was added dropwise followed by dropwise addition of T3P® (≥ 50%w/w in EtOAc; 13.0 mL, 21.3 mmol, 1.30 equiv). The solution was stirred for 5 min before (cyanomethyl)dimethylsulfonium bromide (3.6 g, 19.6 mmol, 1.20 equiv) was added in one portion. The resulting suspension was stirred for 18 h at room temperature and then diluted with CH_2Cl_2. The organic layer was washed with sat. aq. $NaHCO_3$ (1x), water (1x) and brine (1x), and dried over Na_2SO_4. The drying agent was removed by filtration and solvent was removed under reduced pressure. The residue was purified by flash column chromatography (CH_2Cl_2:MeOH 50:1 to 30:1) to give cyanosulfurylide **1** (2.7 g, 13.1 mmol, 80%) as a white foam.

^{1}H NMR (500 MHz, $CDCl_3$) δ 7.89 – 7.81 (m, 2H), 7.51 – 7.35 (m, 3H), 2.87 (s, 6H).

^{13}C NMR (126 MHz, $CDCl_3$) δ 186.30 (CO), 138.33 (C), 131.28 (CH), 128.27 (2xCH), 127.89 (2xCH), 119.60 (CN), 55.20 (C), 28.28 (2xCH_3).

HRMS (ESI): calculated for $[C_{11}H_{12}NOS]^+$: m/z 206.0634 , found : m/z 206.0635.

IR (cm^{-1}, neat): 2165, 1487, 1531, 1361, 1209, 1041, 992.

N-Fmoc-Asp(CSY)-OH 3

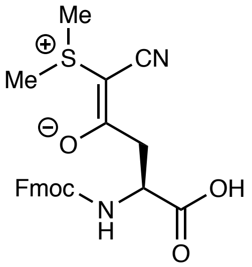

N-Fmoc-Asp(CSY)-O^{t}Bu **5** (1.00 g, 1.93 mmol, 1.00 equiv) was dissolved in CH_2Cl_2 (10 mL) and cooled to 0 °C followed by addition of trifluoroacetic acid (10 mL). The reaction was allowed to warm to room temperature and stirred for 1 h. The solvent was removed under reduced pressure and the resulting oil was diluted with a small volume of CH_2Cl_2 and triturated with Et_2O until no further formation of a solid was observed. The solid was filtered off and washed with Et_2O and residual solvent was removed under reduced pressure to give *N*-Fmoc-Asp(CSY)-OH **3** (0.85 g, 1.89 mmol, 95%) as a powder.

^{1}H NMR (500 MHz, $CDCl_3$) δ 7.78 – 7.72 (m, 2H), 7.64 – 7.56 (m, 2H), 7.39 (tq, *J* = 7.5, 1.0 Hz, 2H), 7.31 (td, *J* = 7.4, 1.2 Hz, 2H), 6.05 (d, *J* = 7.3 Hz, 1H, NH), 4.57 (td, *J* = 7.5, 3.4 Hz, 1H), 4.43 – 4.32 (m, 2H), 4.21 (t, *J* = 7.2 Hz, 1H), 3.35 (dd, *J* = 16.8, 3.4 Hz, 1H), 2.91 (dd, *J* = 16.8, 7.8 Hz, 1H), 2.80 (d, *J* = 9.1 Hz, 6H). (1 acidic proton not observed)

^{13}C NMR (126 MHz, $CDCl_3$) δ 189.95 (CO), 172.99 (CO), 155.96 (CO), 143.97 (2xC), 143.86 (2xC)*, 141.38 (2xC), 127.87 (2xCH), 127.26 (2xCH), 125.30 (2xCH), 120.12 (2xCH), 120.10 (2xCH)*, 116.99 (CN), 67.32 (CH_2), 57.89 (C), 50.56 (CH), 47.22 (CH), 40.60 (CH_2), 28.39 ($2xCH_3$). (* Signals of rotamers)

HRMS (ESI): calculated for $C_{23}H_{22}N_2NaO_5S$ $[M+Na]^+$: 461.1142, found 461.1145.

IR (cm^{-1}, neat): 2177, 1712, 1530, 1258, 1212, 1150, 1043, 908.

$[\alpha]^{26}_D$ (c=0.73, $CHCl_3$): +119.52.

N-Fmoc-Asp(OH)-O^{t}Bu 4

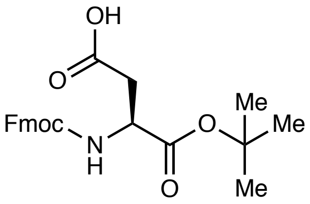

N-Fmoc-Asp(CSY)-O^{t}Bu **5** (50 mg, 0.11 mmol, 1.0 equiv) was dissolved in H_2O/CH_3CN (1:1, v:v, 3 ml) and stirred at room temperature. NCS was added as a solid (29 mg, 0.22 mmol, 2.0 equiv). The reaction was stirred for 5 min. The solution was given into CH_2Cl_2 (40 ml) and washed with aq. 1 N HCl (20 ml), H_2O (20 ml) and brine (20 ml). The organic phase was dried over sodium sulfate and concentrated under reduced pressure. The crude product was purified by flash column chromatography (CH_2Cl_2:MeOH, 10:1 to 9:1) to give **4** (33.4 mg, 0.09 mmol, 80%) as a white solid.

^{1}H NMR (400 MHz, $CDCl_3$) δ 7.76 (d, *J* = 7.5 Hz, 2H), 7.60 (d, *J* = 7.5 Hz, 2H), 7.44 – 7.35 (m, 2H), 7.31 (td, *J* = 7.5, 1.2 Hz, 2H), 5.78 (d, *J* = 8.1 Hz, 1H, NH), 4.55 (dt, *J* = 8.7, 4.6 Hz, 1H), 4.39 (qd, *J* = 10.6, 7.2 Hz, 2H), 4.23 (t, *J* = 7.1 Hz, 1H), 3.12 – 2.81 (m, 2H), 1.47 (s, 9H). (1 acidic proton not observed)

^{13}C NMR (101 MHz, $CDCl_3$) δ 175.41(CO), 169.59 (CO), 156.15 (CO), 143.97 (2xC)*, 143.87 (2xC), 141.44 (2xC), 127.88 (2xCH), 127.23 (2xCH), 125.28 (2xCH), 120.14 (2xCH), 83.13 (C), 67.43 (CH_2), 50.91 (CH), 47.27 (CH), 36.78 (CH_2), 28.00 ($3xCH_3$). (* Signals of rotamers)

HRMS (ESI): calculated for $C_{23}H_{25}NO_6$ $[M+Na]^+$: 434.1574 , found 434.1576.

IR (cm^{-1}, neat): 1711, 1516, 1450, 1370, 1345, 1226, 1153, 1047.

$[\alpha]^{26}_D$ (c=0.29, $CHCl_3$): +9.58.

Chiral HPLC: column: Chiralpak IB (25 × 0.46 cm); eluent: hexanes/2-propanol (8:2) + 0.05% formic acid, flow: 1.0 mL/min; detection: 254 nm. Retention time: t_R = 6.95 min.

N-Fmoc-Asp(CSY)-O^{t}Bu 5

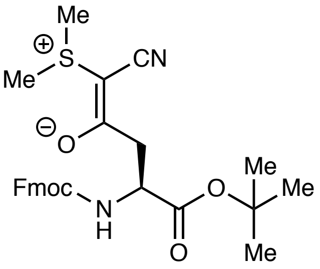

N-Fmoc-Asp(OH)-O^{t}Bu **4** (1.00 g, 2.43 mmol, 1.00 equiv) was dissolved in CH_2Cl_2 (10 mL). *N* ,*N*-Diisopropylethylamine (1.29 mL, 7.29 mmol, 3.00 equiv) was added dropwise followed by dropwise addition of T3P® (≥ 50%w/w in EtOAc; 2.01 mL, 3.16 mmol, 1.30 equiv). The solution was stirred for 5 min before (cyanomethyl)dimethylsulfonium bromide (0.53 g, 2.91 mmol, 1.20 equiv) was added in one portion. The resulting solution was stirred for 18 h at room temperature and then diluted with CH_2Cl_2. The organic layer was washed with sat. aq. $NaHCO_3$ (1x), water (1x) and brine (1x), and dried over Na_2SO_4. The drying agent was removed by filtration and solvent was removed under reduced pressure. The residue was purified by flash column chromatography (CH_2Cl_2:MeOH 40:1 to 30:1) to give *N*-Fmoc-Asp(CSY)-O^{t}Bu **4** (1.07 g, 2.06 mmol, 85%) as a white foam.

^{1}H NMR (500 MHz, $CDCl_3$) δ 7.78 – 7.72 (m, 2H), 7.65 – 7.59 (m, 2H), 7.42 – 7.36 (m, 2H), 7.35 – 7.28 (m, 2H), 5.98 (d, *J* = 9.0 Hz, 1H, NH), 4.55 (dt, *J* = 9.2, 4.5 Hz, 1H), 4.41 (dd, *J* = 10.4, 7.1 Hz, 1H), 4.33 – 4.20 (m, 2H), 3.21 (dd, *J* = 16.2, 5.2 Hz, 1H), 2.91 (dd, *J* = 16.2, 4.2 Hz, 1H), 2.82 (d, *J* = 7.8 Hz, 6H), 1.47 (s, 9H).

^{13}C NMR (126 MHz, $CDCl_3$) δ 189.18 (CO), 170.59 (CO), 156.22 (CO), 144.20 (2xC)*, 144.00 (2xC), 141.37 (2xC)*, 141.35 (2xC), 127.77 (2xCH)*, 127.76 (2xCH), 127.18 (2xCH), 125.43 (2xCH), 125.33 (2xCH)*, 120.04 (2xCH), 118.78 (CN), 82.03 (C), 67.17 (CH_2), 53.30 (C), 51.60 (CH), 47.28 (CH), 40.68 (CH_2), 28.36 (CH_3), 28.21 (CH_3), 28.09 (3xCH_3). (* Signals of rotamers)

HRMS (ESI): calculated for $C_{27}H_{30}N_2NaO_5S$ $[M+Na]^+$: 517.1768, found 517.1764.

IR (cm^{-1}, neat): 2171, 1715, 1589, 1503, 1369, 1248, 1151, 1042.

$[\alpha]^{26}_D$ (c=1.00, $CHCl_3$): +32.30.

Model peptide 6a H-FDGLA-OH

Fmoc—FDGLA—NH_2

Model peptide **6a** was prepared on chloro-trityl resin (loading of 0.38 mmol/g, 500 mg resin). The resin was loaded with Fmoc-Ala-OH according to the general peptide methods. The automated peptide elongation was carried out on a Syro according to general peptide methods. For the peptide cleavage, the peptide was treated with TFA/DODT/H_2O (95:2.5:2.5, v/v) for 2 h and the resin was removed by filtration. The solution was concentrated under reduced pressure and triturated with Et_2O and centrifuged to obtain crude peptide **6a**. The crude peptide was redissolved in H_2O/CH_3CN (1:1, v/v) and purified by preparative HPLC (Method A). The purified peptide was obtained as a white solid (36%, 35 mg).

HRMS (ESI): calculated for $[C_{24}H_{35}N_5O_8]^+$: m/z 522.2558 , found : m/z 522.2554.

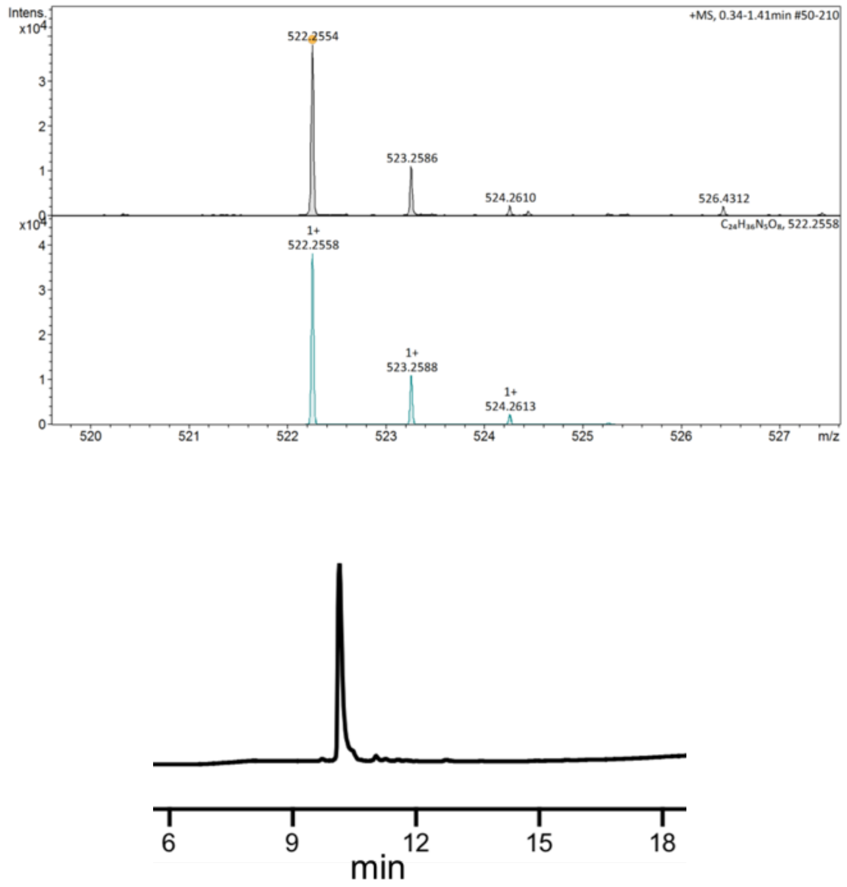

Supplementary Figure 14. **HRMS and HPLC trace of peptide 6a.**

Model peptide 6b H-FD(CSY)GLA-OH

Fmoc—FD(CSY)GLA—NH_2

Model peptide **6b** was prepared on chloro-trityl resin (loading of 0.40 mmol/g, 500 mg resin). The resin was loaded with Fmoc-Ala-OH according to the general peptide methods. The automated peptide elongation was carried out on a Syro according to general peptide methods. Fmoc-Asp(CSY)-OH **3** was coupled manually (2 equiv, 60min, one coupling). For the peptide cleavage, the peptide was treated with TFA/DODT/H_2O (95:2.5:2.5, v/v) for 2 h and the resin was removed by filtration. The solution was concentrated under reduced pressure and triturated with Et_2O and centrifuged to obtain crude peptide **6b**. The crude peptide was redissolved in H_2O/CH_3CN (1:1, v/v) and purified by preparative HPLC (Method A). The purified peptide was obtained as a white solid (41%, 51 mg).
HRMS (ESI): calculated for $[C_{28}H_{41}N_6O_7S]^+$: m/z 605.2752 , found : m/z 605.2743.

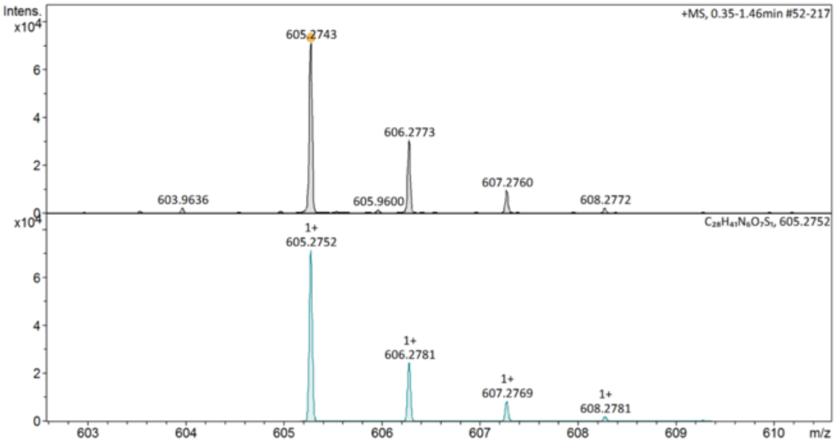

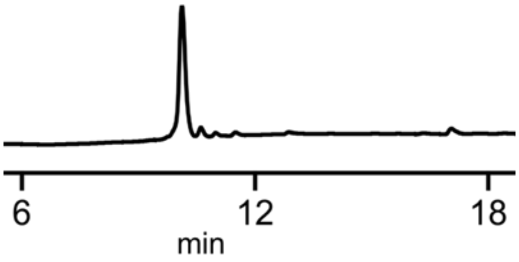

Supplementary Figure 15. **HRMS and HPLC trace of peptide 6b.**

Model peptide 7 Fmoc-KWRC(S*t*Bu)FD(CSY)GLA-OH

Fmoc—KWRC(S^{t}Bu)FD(CSY)GLA—OH

Model peptide **7** was prepared on chloro-trityl resin (loading of 0.35 mmol/g, 1000 mg resin). The resin was loaded with Fmoc-Ala-OH according to the general peptide methods. The automated peptide elongation was carried out on Syro according to general peptide methods. Fmoc-Asp(CSY)-OH **3** was coupled manually (2 equiv, 60 min, one coupling). Fmoc-Cys(S^{t}Bu)-OH was coupled manually (2 equiv, 2 equiv DIC, 2 equiv Cl-HOBt, 120 min, one coupling). For the peptide cleavage, the peptide was treated with TFA/DODT/H_2O (95:2.5:2.5, v/v) for 2 h and the resin was removed by filtration. The solution was concentrated under reduced pressure and triturated with Et_2O and centrifuged to obtain crude peptide **7**. The crude peptide was redissolved in H_2O/CH_3CN (1:1, v/v) and purified by preparative HPLC (Method A). The purified peptide was obtained as a white solid (32%, 166 mg).

HRMS (ESI): calculated for $[C_{73}H_{99}N_{15}O_{13}S_3]^{2+}$: m/z 744.8349 , found : m/z 744.8348.

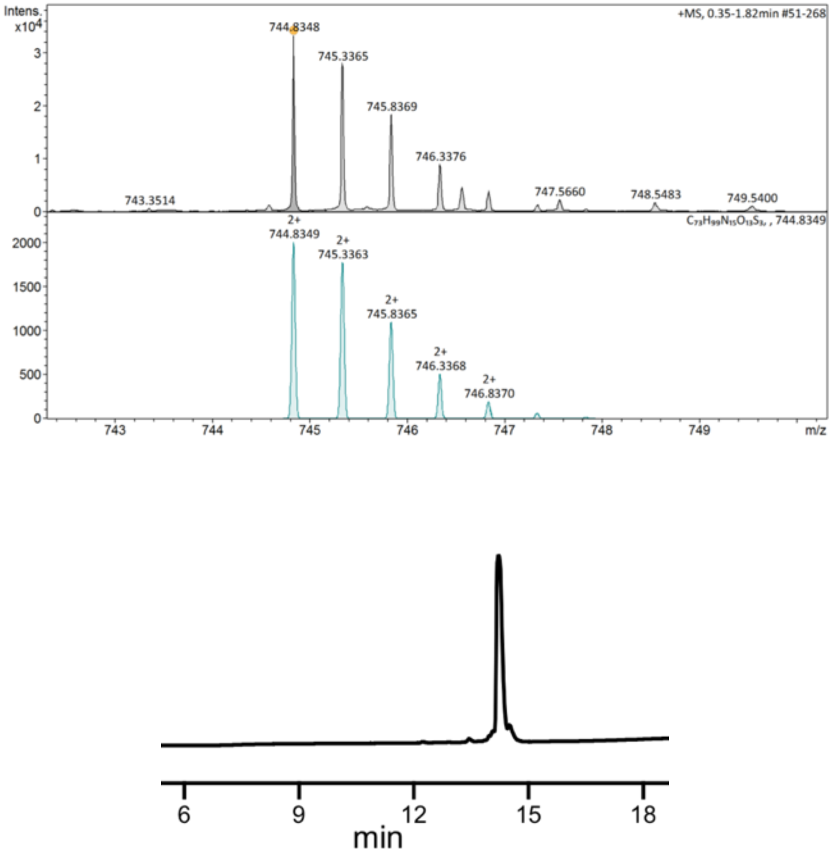

Supplementary Figure 16. **HRMS and HPLC trace of peptide 7.**

Model peptide 8 Fmoc-KWRC(S*t*Bu)FDGLA-OH

Fmoc—KWRC(S^{t}Bu)FDGLA—OH

Model peptide **7** (10.0 mg, 6.8 μmol) either purified or after global resin cleavage, was dissolved in aqueous NaOAc/AcOH (pH 4.5, 200 mM) or non-buffered acidic water (pH 3.0, 400 mM NaCl) containing 10% CH_3CN (peptide concentration 1 mM). Once peptide **7** was fully dissolved, NCS was added from a stock solution in CH_3CN (100 mM) in portions to avoid oxidation of other amino acid moieties (portions of 0.5 equiv). The reaction was monitored by mass spectroscopy and analytical HPLC (note: the cyanosulfurylides absorb strongly at 254 nm; cleavage results in a decrease in signal). Once full conversion was indicated the crude peptide was purified by HPLC (Method A) and was obtained as a white solid after lyophilization (80%, 7.5 mg, 5.4 μmol).

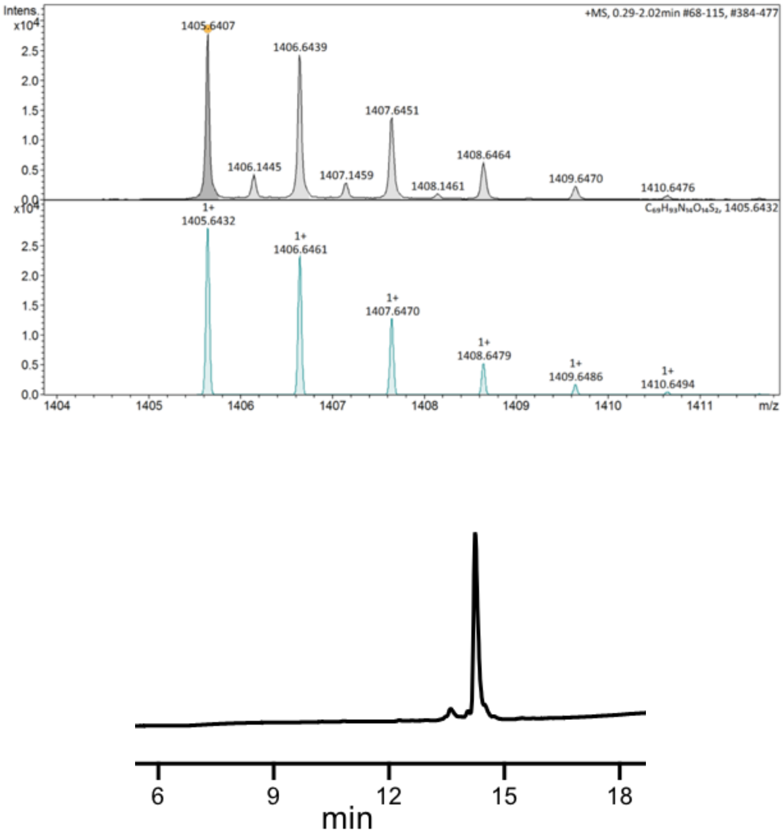

Supplementary Figure 17. **HRMS and HPLC trace of peptide 8.**

Model peptide S2 Fmoc-KWRCFDGLA-NH_2

Fmoc—KWRCFDGLA—NH_2

Model peptide **S2** was prepared on Rink-amide resin (loading of 0.39 mmol/g, 500 mg resin). The resin was loaded with Fmoc-Ala-OH according to the general peptide methods. The automated peptide elongation was carried out on Syro according to general peptide methods. Fmoc-Asp(CSY)-OH **3** was coupled manually (2 equiv, 60 min, one coupling). Fmoc-Cys(Trt)-OH was coupled manually (2 equiv, 2 equiv DIC, 2 equiv Cl-HOBt, 120 min, one coupling). After the last coupling, the resin was swollen in CH_2Cl_2 and incubated in DMF/H_2O/HFIP (90:8:2) with NCS (from 1.5 equiv, depending on resin and peptide the amount can vary but should be added in portions) for 2 min and washed with CH_2Cl_2 for three times. The reaction was monitored by mass spectroscopy and analytical HPLC (note: the cyanosulfurylides absorb strongly at 254 nm; cleavage results in a decrease in signal). An increasing amount of water is beneficial for the successful deprotection. For the peptide cleavage, the peptide was treated with TFA/DODT/H_2O (95:2.5:2.5, v/v) for 2 h and the resin was removed by filtration. The solution was concentrated under reduced pressure and triturated with Et_2O and centrifuged to obtain crude peptide **S1**. The crude peptide was redissolved in H_2O/CH_3CN (1:1, v/v) and purified by preparative HPLC (Method A). The purified peptide was obtained as a white solid (26%, 74 mg).

HRMS (MALDI): calculated for $[C_{65}H_{85}N_{15}O_{13}S]^+$: m/z 1316.6245 , found : m/z 1316.6344.

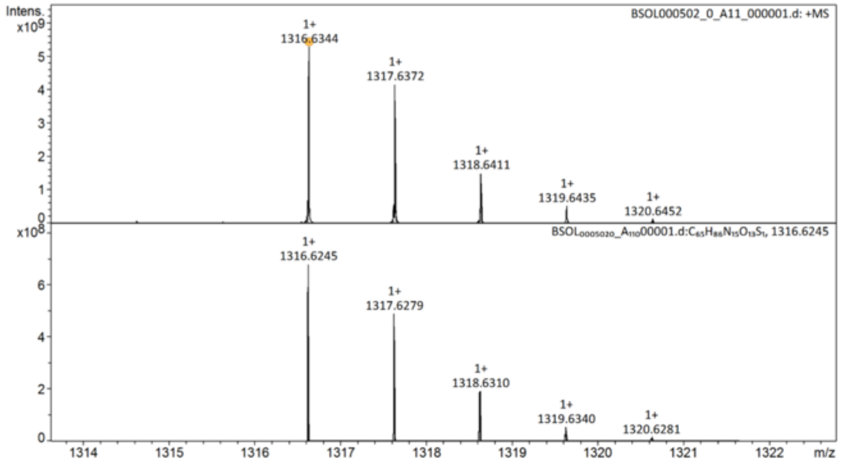

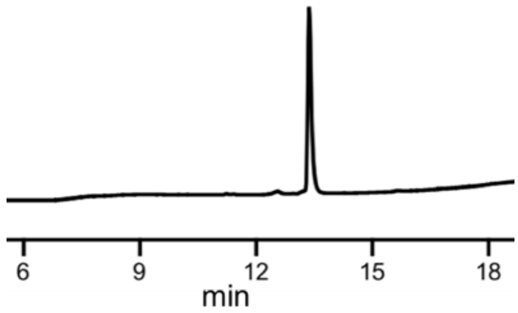

Supplementary Figure 18. **HRMS and HPLC trace of peptide S2.**

Model peptide S5-L H-VYPDGA-OH

H—VYPDGA—OH

Model peptide **S5-L** was prepared on chloro-trityl resin (loading of 0.45 mmol/g, 250 mg resin). The resin was loaded with Fmoc-Ala-OH according to the general peptide methods. The automated peptide elongation was carried out on a Syro according to general peptide methods. For the peptide cleavage, the peptide was treated with TFA/DODT/H_2O (95:2.5:2.5, v/v) for 2 h and the resin was removed by filtration. The solution was concentrated under reduced pressure and triturated with Et_2O and centrifuged to obtain crude peptide **S5-L**. The crude peptide was redissolved in H_2O/CH_3CN (1:1, v/v) and purified by preparative HPLC (Method D). The purified peptide was obtained as a white solid (33%, 23 mg).

HRMS (ESI): calculated for $[C_{28}H_{41}N_6O_{10}]^+$: m/z 621.2879 , found : m/z 621.2873.

Model peptide S5-D H-VYPDGA-OH

H—VYPDGA—OH

Model peptide **S5-D** was prepared on chloro-trityl resin (loading of 0.45 mmol/g, 250 mg resin). The resin was loaded with Fmoc-Ala-OH according to the general peptide methods. The automated peptide elongation was carried out on a Syro according to general peptide methods. Fmoc-D-Asp(OtBu)OH was coupled manually (2 equiv, 60 min, one coupling). For the peptide cleavage, the peptide was treated with TFA/DODT/H_2O (95:2.5:2.5, v/v) for 2 h and the resin was removed by filtration. The solution was concentrated under reduced pressure and triturated with Et_2O and centrifuged to obtain crude peptide **S5-D**. The crude peptide was redissolved in H_2O/CH_3CN (1:1, v/v) and purified by preparative HPLC (Method D). The purified peptide was obtained as a white solid (25%, 17 mg).

HRMS (ESI): calculated for $[C_{28}H_{41}N_6O_{10}]^+$: m/z 621.2879 , found : m/z 621.2878.

Model peptide iso-S6 H-VYPDGA-OH

H—VYPD(iso)GA—OH

Model peptide **iso-S6** was prepared on chloro-trityl resin (loading of 0.45 mmol/g, 250 mg resin). The resin was loaded with Fmoc-Ala-OH according to the general peptide methods. The automated peptide elongation was carried out on a Syro according to general peptide methods. Fmoc-L-Asp(OH)OtBu was coupled manually (2 equiv, 60 min, one coupling). For the peptide cleavage, the peptide was treated with TFA/DODT/H_2O (95:2.5:2.5, v/v) for 2 h and the resin was removed by filtration. The solution was concentrated under reduced pressure and triturated with Et_2O and centrifuged to obtain crude peptide **iso-S6**. The crude peptide was redissolved in H_2O/CH_3CN (1:1, v/v) and purified by preparative HPLC (Method D). The purified peptide was obtained as a white solid (30%, 20 mg).

HRMS (ESI): calculated for $[C_{28}H_{41}N_6O_{10}]^+$: m/z 621.2879 , found : m/z 621.2867.

Model peptide AspI-S7 H-VYPDGA-OH

H—VYPD(Imide)GA—OH

Model peptide **AspI-S7** was prepared by incubation of resin-bound **S5-L** in 20% piperidine in DMF for 1h. For the peptide cleavage, the peptide was treated with TFA/DODT/H_2O (95:2.5:2.5, v/v) for 2 h and the resin was removed by filtration. The solution was concentrated under reduced pressure and triturated with Et_2O and centrifuged to obtain crude peptide **iso-S6**. The crude peptide was redissolved in H_2O/CH_3CN (1:1, v/v) and purified by preparative HPLC (Method D). The purified peptide was obtained as a white solid (analytical scale, no yield determined).

HRMS (ESI): calculated for $[C_{28}H_{40}N_6O_9]^+$: m/z 603.2773 , found : m/z 603.2765.

Model peptide CSY-S8 H-VYPD(CSY)GA-OH

H—VYPD(CSY)GA—OH

Model peptide **CSY-S8** was prepared on chloro-trityl resin (loading of 0.45 mmol/g, 250 mg resin). The resin was loaded with Fmoc-Ala-OH according to the general peptide methods. The automated peptide elongation was carried out on a Syro according to general peptide methods. Fmoc-L-Asp(CSY)OH was coupled manually (2 equiv, 60 min, one coupling). For the peptide cleavage, the peptide was treated with TFA/DODT/H_2O (95:2.5:2.5, v/v) for 2 h and the resin was removed by filtration. The solution was concentrated under reduced pressure and triturated with Et_2O and centrifuged to obtain crude peptide **iso-S6**. The crude peptide was redissolved in H_2O/CH_3CN (1:1, v/v) and purified by preparative HPLC (Method D). The purified peptide was obtained as a white solid (35%, 28 mg).

HRMS (ESI): calculated for $[C_{32}H_{47}N_7O_9]^+$: m/z 704.3072 , found : m/z 704.3067.

Teduglutide 9

H—HGDGSFSDEM[10]NTILDNLAAR[20]DFINWLIQTK[30]ITD—OH

Purified teduglutide(CSY) **10** (22.0 mg, 5.6 μmol) was dissolved in aqueous NaOAc/AcOH (pH 4.5, 200 mM) or acidic saline (pH 3.0, 400 mM NaCl) containing 20% CH_3CN (peptide concentration 1 mM). Once teduglutide(CSY) **10** was fully dissolved, NCS was added from a stock solution in CH_3CN (100 mM) in portions to avoid oxidation of other amino acid moieties (portions of 0.55 equiv). The reaction was monitored by mass spectroscopy and analytical HPLC (note: the cyanosulfurylides absorb strongly at 254 nm; cleavage results in a decrease in signal). Once full conversion was indicated the crude peptide was purified by HPLC (Method A) and teduglutide **9** was obtained as a white solid after lyophilization (79%, 17.0 mg, 4.4 μmol).

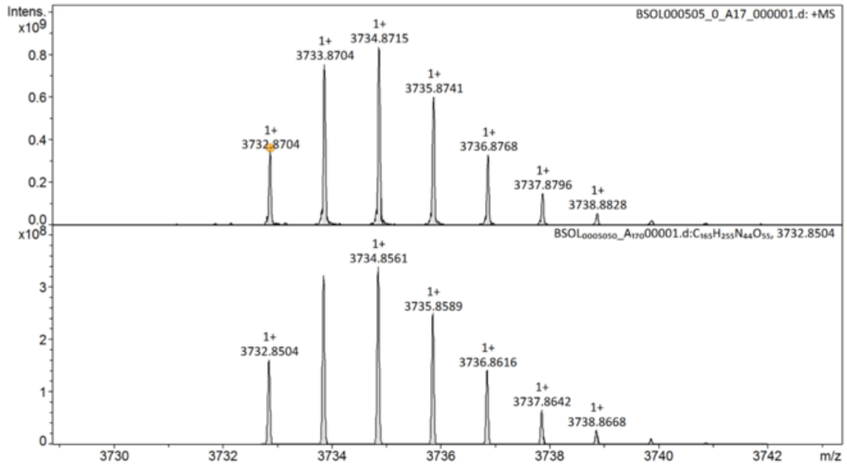

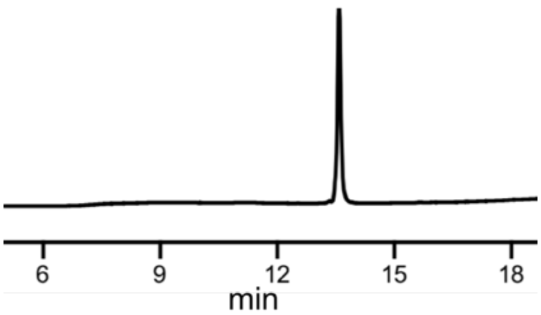

Supplementary Figure 19. **HRMS and HPLC trace of peptide 9.**

Teduglutide(CSY) 10

H—HGD(CSY)GSFSDEM[10]NTILD(CSY)NLAAR[20]DFINWLIQTK[30]ITD—OH

Teduglutide(CSY) **10** was prepared on chloro-trityl resin (loading of 0.34 mmol/g, 500 mg resin). The resin was loaded with Fmoc-Asp(O^{t}Bu)-OH according to the general peptide methods. The automated peptide elongation was carried out on a Syro according to general peptide methods. Fmoc-Asp(CSY)-OH **3** was coupled manually (2 equiv, 60 min, one coupling). For the peptide cleavage, the peptide was treated with TFA/DODT/H_2O (95:2.5:2.5, v/v) for 2 h and the resin was removed by filtration. The solution was concentrated under reduced pressure and triturated with Et_2O and centrifuged to obtain crude teduglutide(CSY) **10**. The crude peptide was redissolved in H_2O/CH_3CN (1:1, v/v) and purified by preparative HPLC (Method A). The purified peptide was obtained as a white solid (34%, 225 mg).

HRMS (ESI): calculated for $[C_{173}H_{264}N_{46}O_{53}S_2]^+$: m/z 3897.8818 , found : m/z 3897.8904.

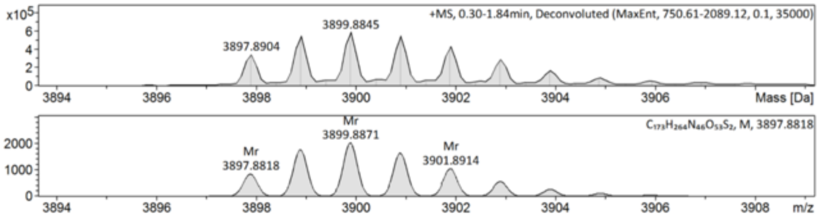

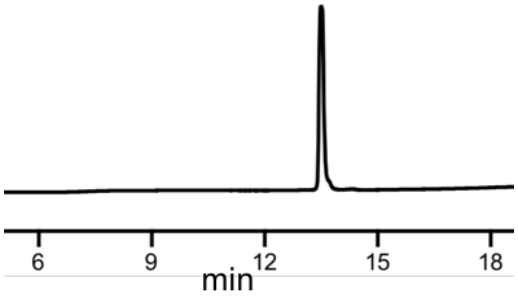

Supplementary Figure 20. **HRMS and HPLC trace of peptide 10.**

Folding of LDLa 11

LDLa **11** (1 mg, 0.3 μmol) was folded according to a reported procedure.[2] Briefly, the peptide was dissolved (150 μg mL^{-1}) in refolding buffer at pH 8.5 containing of GSH (3 mM), GSSG (0.3 mM), Tris-HCl (50 mM), NaCl (150 mM) and $CaCl_2$ (2.5 mM). Folding was monitored with HPLC for 15 h. The folded LDLa was purified by preparative HPLC. After lyophilizing the product was obtained as a white solid (36%, 0.4 mg).

HRMS (ESI): calculated for $[C_{180}H_{274}N_{51}O_{64}S_6]^{3+}$: m/z 1455.2874 , found : m/z 1455.2682.

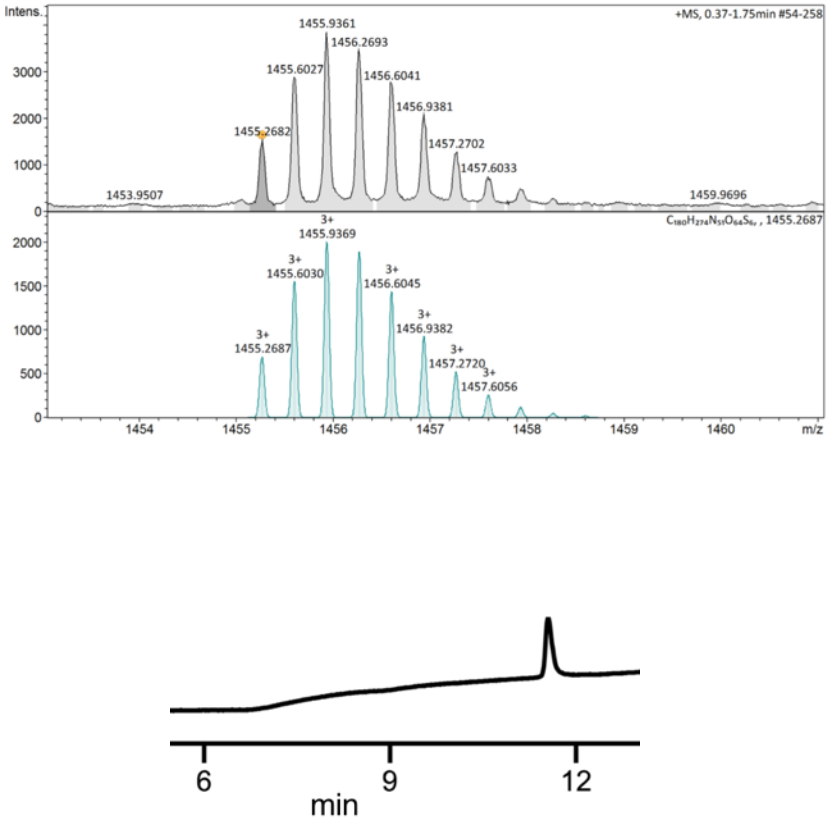

Supplementary Figure 21. **HRMS and HPLC trace of peptide 11.**

LDLa(CSY/SS^{t}Bu) 12

H—ZDVKC(S^{t}Bu)SLGYF^{10}PC(S^{t}Bu)GNITKC(S^{t}Bu)LP^{20}QLLHC(S^{t}Bu)NGVD(CSY)D(CSY)^{30}C(S^{t}Bu)GNQAD(CSY)ED(CSY)NC(S^{t}Bu)^{40}G—OH

LDLa(CSY/SS^{t}Bu) **12** was prepared on chloro-trityl resin (loading of 0.28 mmol/g, 1 g resin). The resin was loaded with Fmoc-Gly-OH according to the general peptide methods. The automated peptide elongation was carried out on Syro according to general peptide methods. Fmoc-Asp(CSY)-OH **3** was coupled manually (2 equiv, 60 min, one coupling). Fmoc-Cys(SS^{t}Bu)-OH was coupled manually (2 equiv, 2 equiv DIC, 2 equiv Cl-HOBt, 120 min, one coupling). The final pyroglutamic acid was coupled unprotected. For the peptide cleavage, the peptide was treated with TFA/DODT/H_2O (95:2.5:2.5, v/v) for 2 h and the resin was removed by filtration. The solution was concentrated under reduced pressure and triturated with Et_2O and centrifuged to obtain crude peptide **7**. The crude peptide was redissolved in H_2O/CH_3CN (1:1, v/v) and purified by preparative HPLC (Method B). LDLa(CSY/SS^{t}Bu) **12** was obtained as a white solid (14%, 212.4 mg).

HRMS (ESI): calculated for $[C_{220}H_{348}N_{55}O_{60}S_{16}]^{3+}$: m/z 1744.0462 , found : m/z 1744.0448.

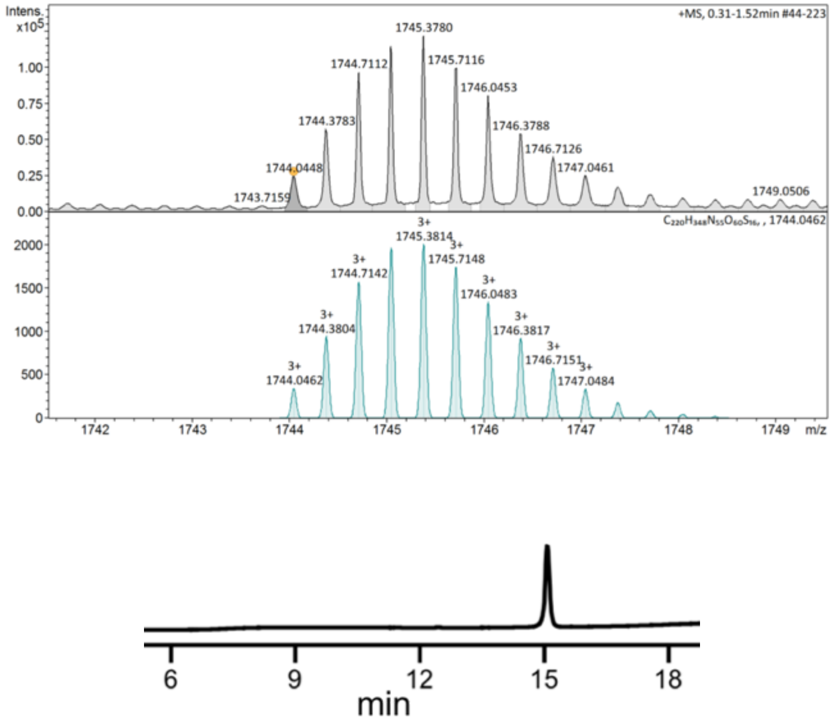

Supplementary Figure 22. **HRMS and HPLC trace of peptide 12.**

LDLa(SS^{t}Bu) 13

H—ZDVKC(S^{t}Bu)SLGYF^{10}PC(S^{t}Bu)GNITKC(S^{t}Bu)LP^{20}QLLHC(S^{t}Bu)NGVDD^{30}C(S^{t}Bu)GNQADEDNC(S^{t}Bu)^{40}G—OH

LDLa(CSY/SS^{t}Bu) **12** (10.0 mg, 1.9 μmol) was dissolved in acidic saline (pH 3.0, 400 mM NaCl) containing 25% CH_3CN (peptide concentration 1 mM). Once fully dissolved, NCS was added from a stock solution in CH_3CN (100 mM) in portions to avoid oxidation of other amino acid moieties (portions of 0.55 equiv). The reaction was monitored by mass spectroscopy and analytical HPLC (cyanosulfurylides absorb strongly at 254 nm; cleavage results in a decrease in signal). Once full conversion was indicated the crude peptide was purified by HPLC (Method B) and LDLa(SS^{t}Bu) **13** obtained as a white solid after lyophilization (75%, 6.3 mg, 1.4 μmol).

HRMS (ESI): calculated for $[C_{204}H_{328}N_{51}O_{64}S_{12}]^{3+}$: m/z 1633.3537 , found : m/z 1633.3530.

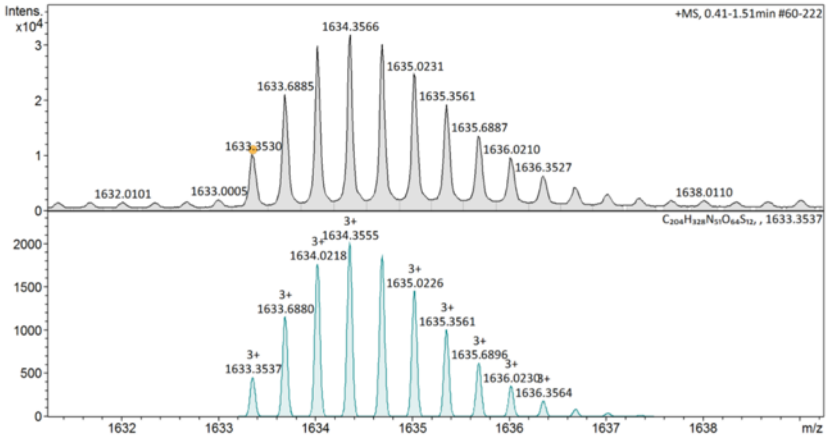

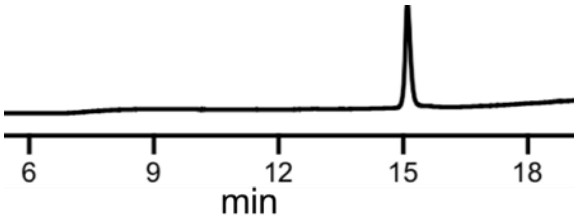

Supplementary Figure 23. **HRMS and HPLC trace of peptide 13.**

LDLa 14

H—ZDVKCSLGYF^{10}PCGNITKCLP^{20}QLLHCNGVDD^{30}CGNQADEDNC^{40}G—OH

LDLa(SS^{t}Bu) **13** (2.5 mg, 1.0 μmol) was dissolved in PBS/CH_3CN (9:1, pH 7.0, TCEP 20 mM, peptide concentration 250 μM) and heated to 50 °C and stirred overnight. The conversion was monitored by mass spectroscopy and analytical HPLC. The crude peptide was purified by preparative HPLC (Method A) and lyophilized. LDLa **11** was obtained as a white solid (55%, 1.2 mg) and stored under argon atmosphere at -20°C. HRMS (ESI): calculated for $[C_{180}H_{280}N_{51}O_{64}S_6]^{3+}$: m/z 1457.2844 , found : m/z 1457.2847.

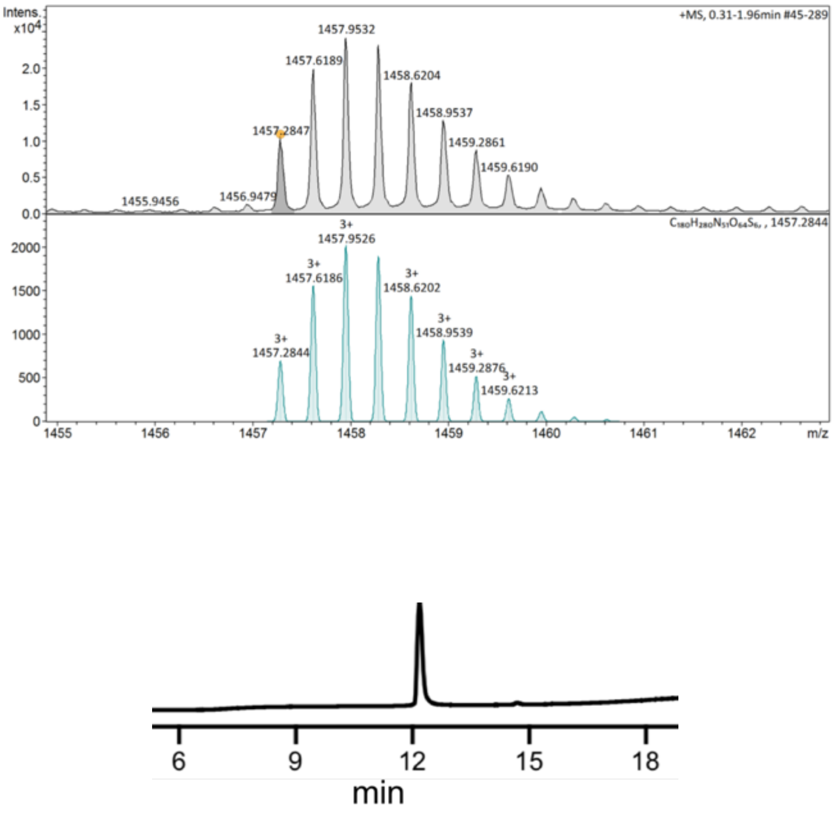

Supplementary Figure 24. **HRMS and HPLC trace of peptide 14.**

Ubiquitin Segment 16

Ubiquitin Segment **16** was prepared on chloro-trityl resin (loading of 0.4 mmol/g, 250 mg resin). The resin was loaded with Fmoc-Gly-OH according to the general peptide methods. The automated peptide elongation was carried out on a Syro according to general peptide methods. Fmoc-Asp(CSY)-OH **3** was coupled manually (2 equiv, 60 min, one coupling). Fmoc-photoOpr-OH[3] was coupled manually (2 equiv, 180 min, one coupling). For peptide cleavage, the peptide was treated with TFA/DODT/H2O (95:2.5:2.5, v/v) for 2 h and the resin was removed by filtration. The solution was concentrated under reduced pressure and triturated with Et_2O and centrifuged to obtain crude Ubiquitin Segment **15**. The crude peptide was redissolved in H_2O/CH_3CN (1:1, v/v) and purified by preparative HPLC (Method C) to give photo-Ubiquitin **15**. The product containing fractions were irradiated for 10 minutes at 365 nm. Progress was measured using analytical HPLC. After completion, the solution was lyophilized to give the deprotected peptide as an off-white solid (4%, 11 mg).

HRMS (ESI): calculated for photo-protected Ubiquitin Segment **15** $[C_{139}H_{222}N_{42}O_{44}S]^+$: m/z 3215.6146 , found : m/z 3215.6079.

calculated for Ubiquitin Segment **16** $[C_{130}H_{215}N_{41}O_{40}S]^+$: m/z 3022.5771 , found : m/z 3022.5831.

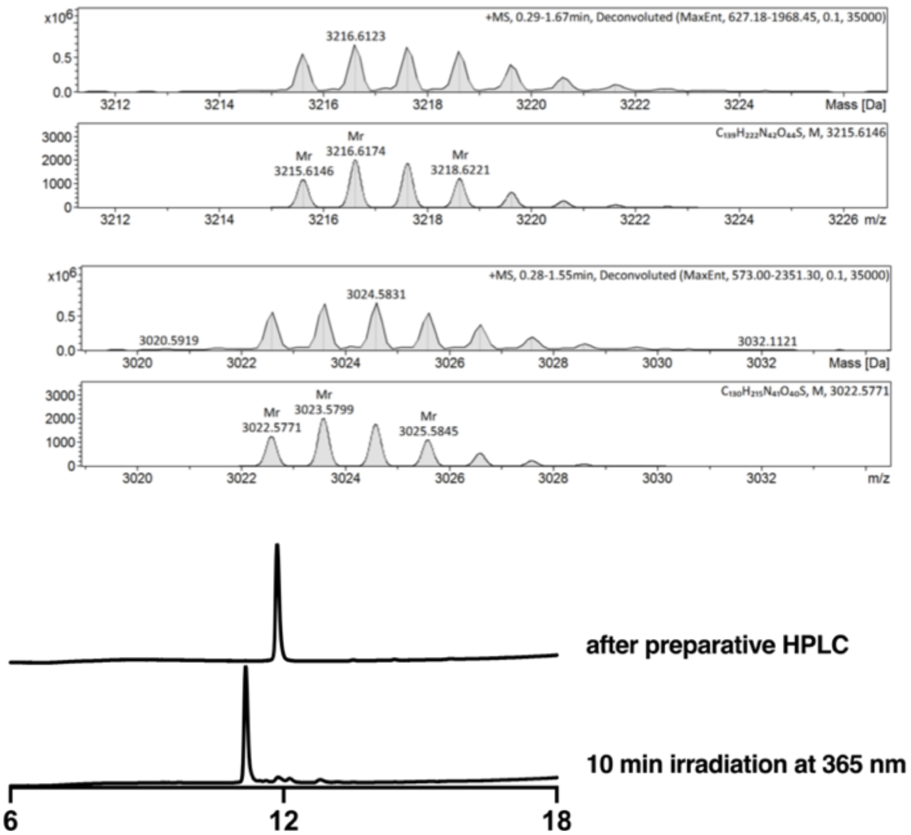

Supplementary Figure 25. **HRMS and HPLC trace of peptide 16.**

Ubiquitin Segment 17

Ubiquitin Segment **17** was prepared on Rink-amide resin (loading of 0.250 mmol/g, 320 mg resin). The resin was loaded with Fmoc-Leu-α-ketoacid(acid labile)[4] according to the general peptide methods. The automated peptide elongation was carried out on a Syro according to general peptide methods. For the peptide cleavage, the peptide was treated with TFA/DODT/H_2O (95:2.5:2.5, v/v) for 2 h and the resin was removed by filtration. The solution was concentrated under reduced pressure and triturated with Et_2O and centrifuged to obtain crude Ubiquitin Segment **17**. The crude peptide was redissolved in H_2O/CH_3CN (1:1, v/v) and purified by preparative HPLC (Method C). The purified peptide was obtained as a white solid (15%, 62 mg).

HRMS (ESI): calculated for $[C_{253}H_{421}N_{65}O_{78}]^+$: m/z 5618.0975 , found : m/z 5618.0896.

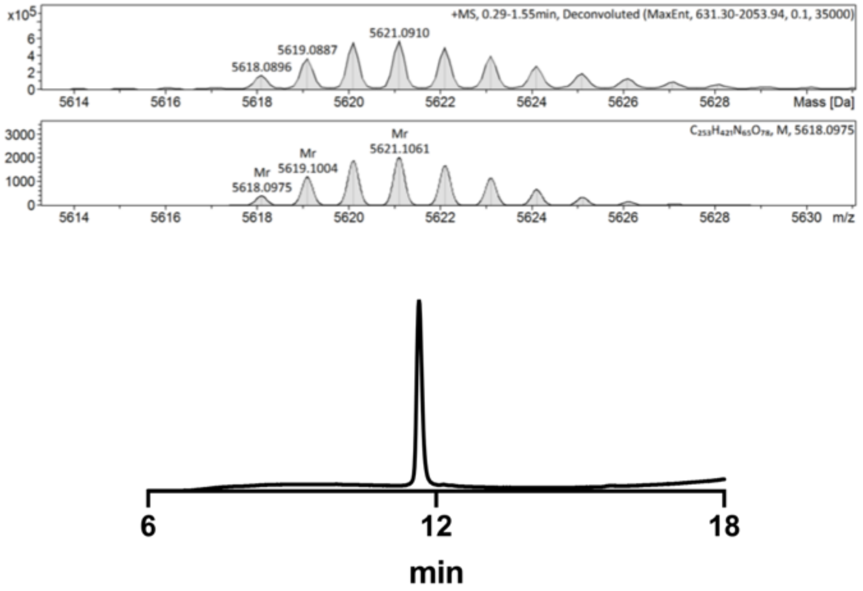

Supplementary Figure 26. **HRMS and HPLC trace of peptide 17.**

Ubiquitin(E51Hse/Asp52Asp(CSY)) 19

Ubiquitin Segment **16** (11.7 mg, 2.08 µmol, 1.5 equiv) and ubiquitin Segment **17** (4.2 mg, 1.39 µmol) were mixed with HFIP/AcOH (62 µL, 22.5 mM, 1:1, v/v, 1 vol% H_2O) at 45 ºC and shaken. The progress of the reaction was monitored by analytical HPLC. After 3 h the reaction was deemed complete and diluted with H_2O/CH_3CN (1:1, v/v, 0.1 vol% TFA) and purified by preparative HPLC. The purified *depsi*-ubiquitin **18** was obtained as a white solid (3.4 mg, 28%).

HRMS (ESI): calculated for $[C_{382}H_{636}N_{106}O_{116}S]^+$: m/z 8596.6847 , found : m/z 8597.7065.

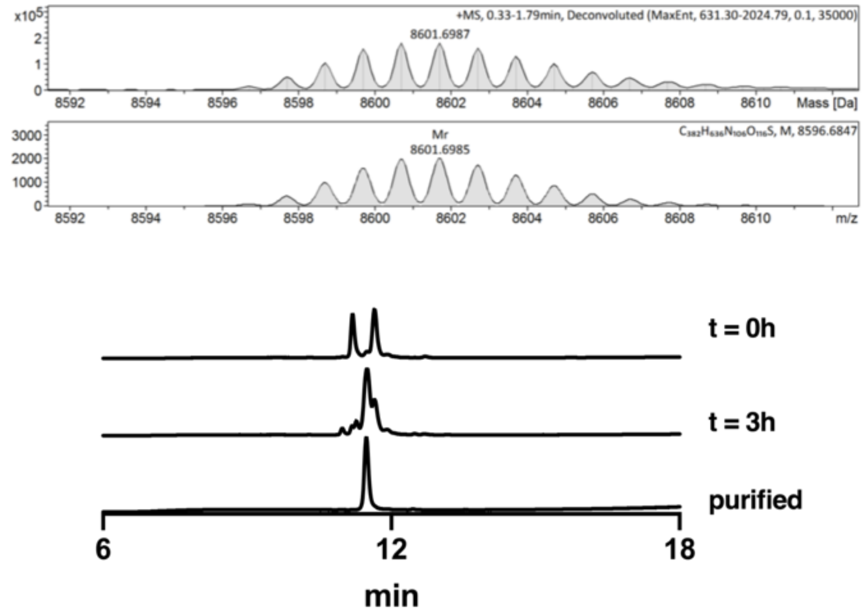

Supplementary Figure 27. **HRMS and HPLC trace of peptide 18.**

depsi-Ubiquitin **18** (2 mg) was dissolved in rearrangement buffer (200 µL, 6 M guanidinium hydrochloride, 200 mM sodium carbonate, pH 9.5) and shaken at room temperature. After 2 h the mixture was given to the folding buffer (10 mL, 50 mM Tris, 150 mM NaCl, pH 7.5) and dialyzed against the folding buffer at 4 ºC. Folded ubiquitin(E51Hse/Asp52Asp(CSY)) **19** (0.9 mg, 45%) was concentrated and stored in aliquots at –80 ºC.

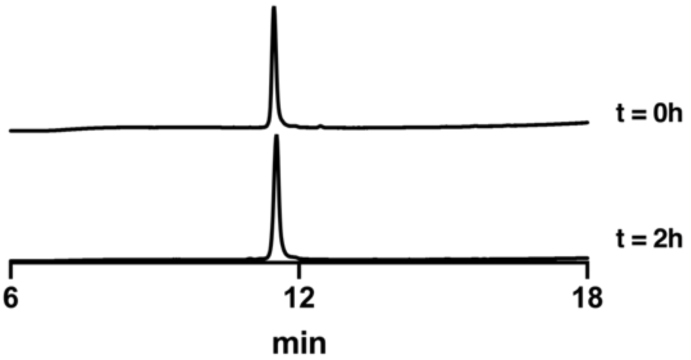

Supplementary Figure 28. **HPLC trace of peptide 19.**

Ubiquitin(E51Hse) 20

Ubiquitin(E51Hse/Asp52Asp(CSY)) 19 (30 µg, 3.5 nmol) in acidic saline (100 µL, 200 mM NaCl pH 3.0) was titrated with NCS from a CH_3CN stock (10 mM). NCS was added from a stock solution in CH_3CN (100 mM) in portions to avoid oxidation of other amino acid moieties (1.4 equiv in total; portions of 0.4 equiv and 0.2 equiv). The reaction was monitored by mass spectroscopy and analytical HPLC (note: the cyanosulfurylides absorb strongly at 254 nm; cleavage results in a decrease in signal). Once full conversion was indicated succinimide was removed by spin-filtration against acidic saline to obtain **ubiquitin(E51Hse) 20** and used for analysis without further purification.

HRMS (ESI): calculated for $[C_{378}H_{631}N_{105}O_{117}]^+$: m/z 8513.6654 , found : m/z 8514.6357.

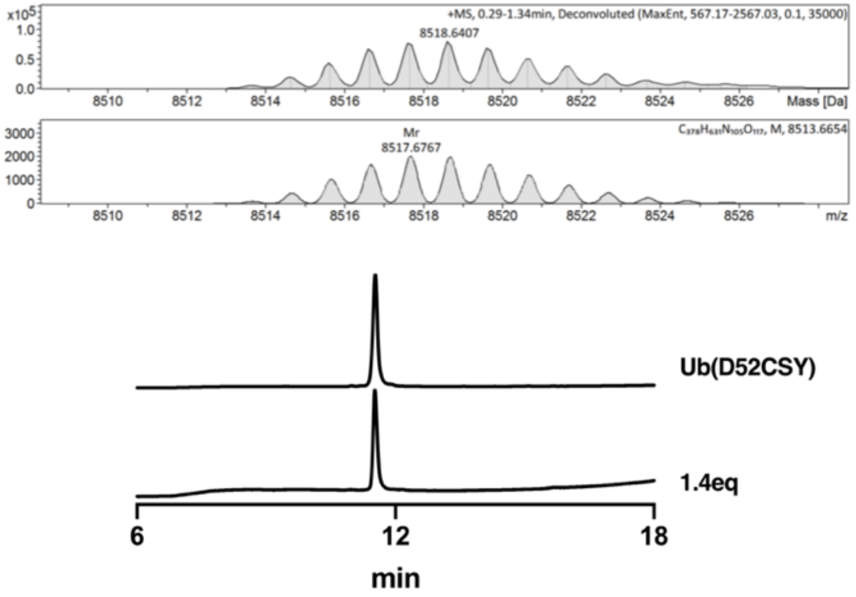

Supplementary Figure 29. **HRMS and HPLC trace of peptide 20.**

Cyanosulfurylide 1

^{1}H NMR

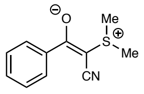

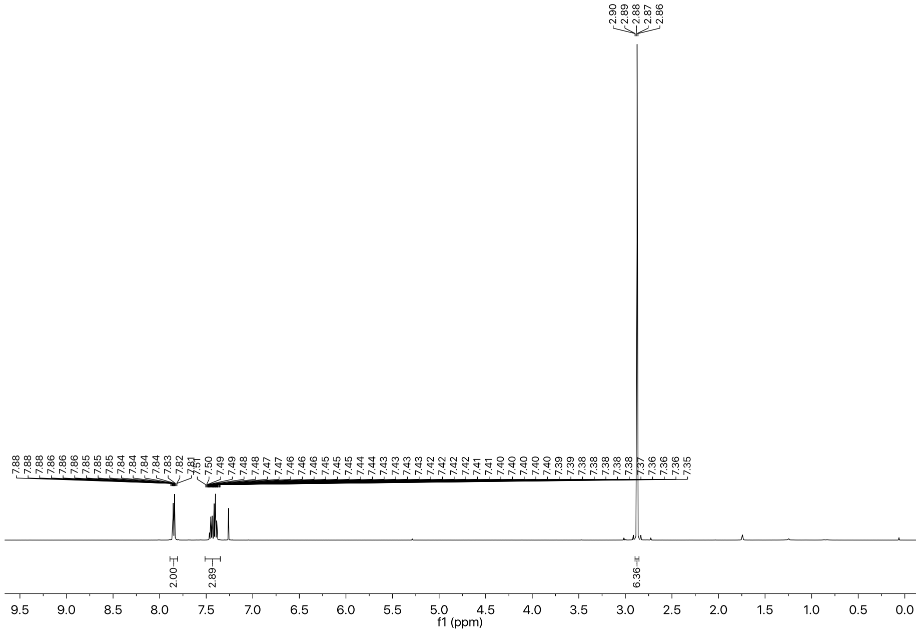

^{13}C NMR

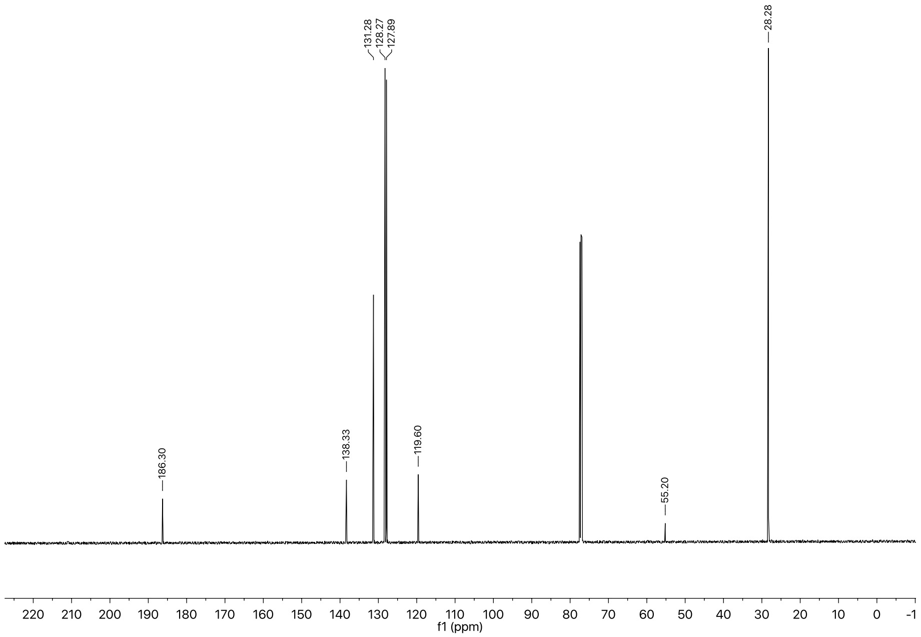

Supplementary Figure 30 **^{1}H and ^{13}C NMR spectra of compound 1.**

N-Fmoc-Asp(CSY)-OH 3

^{1}H NMR

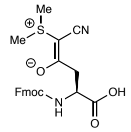

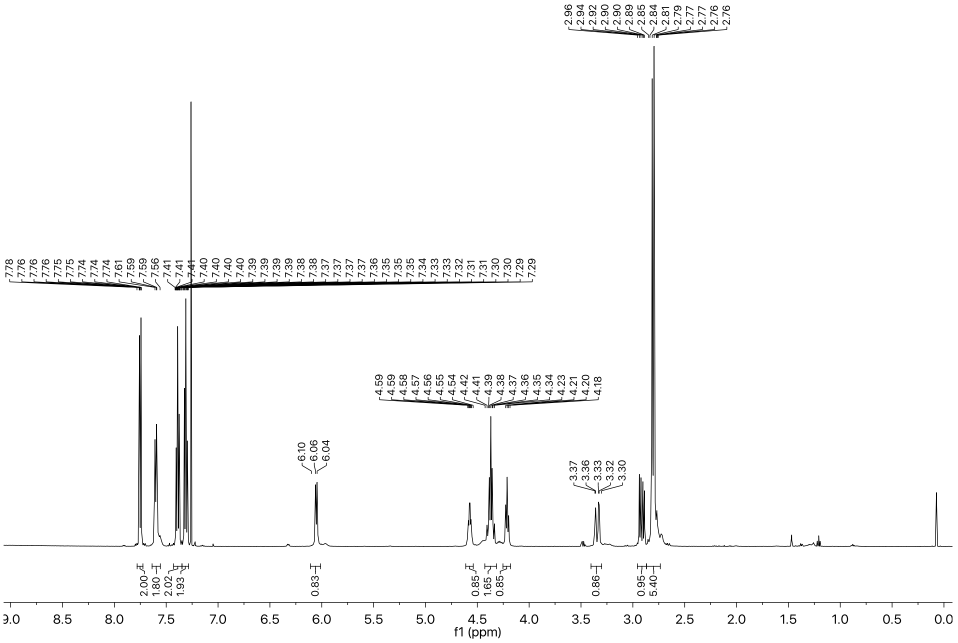

^{13}C NMR

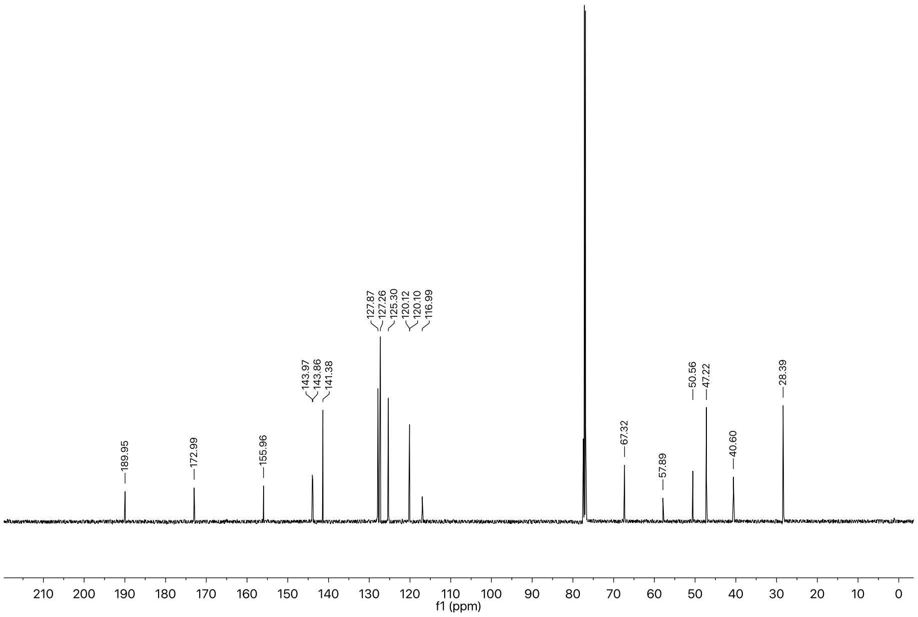

Supplementary Figure 31. **^{1}H and ^{13}C NMR spectra of compound 3.**

N-Fmoc-Asp(OH)-O*t*Bu 4

^{1}H NMR

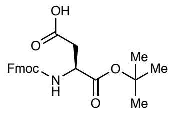

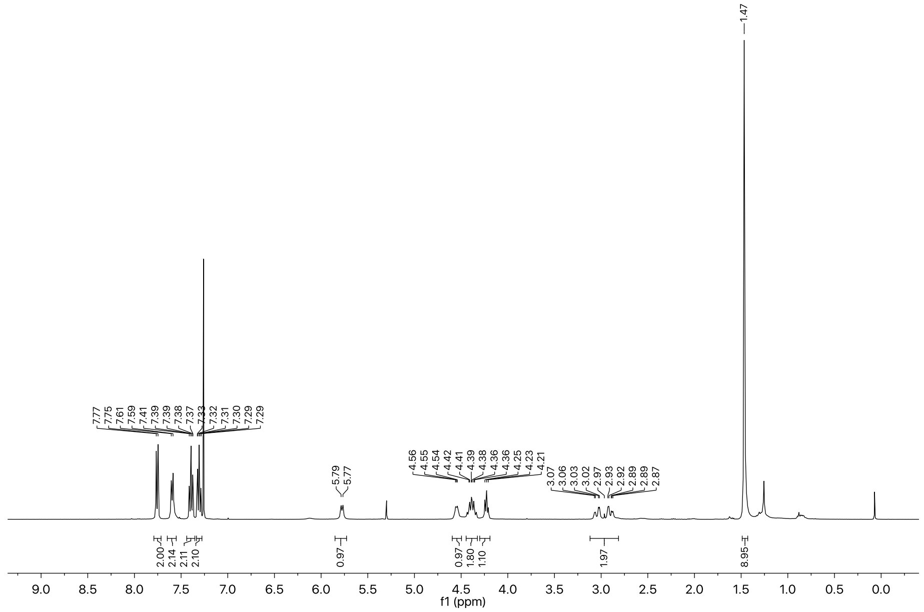

^{13}C NMR

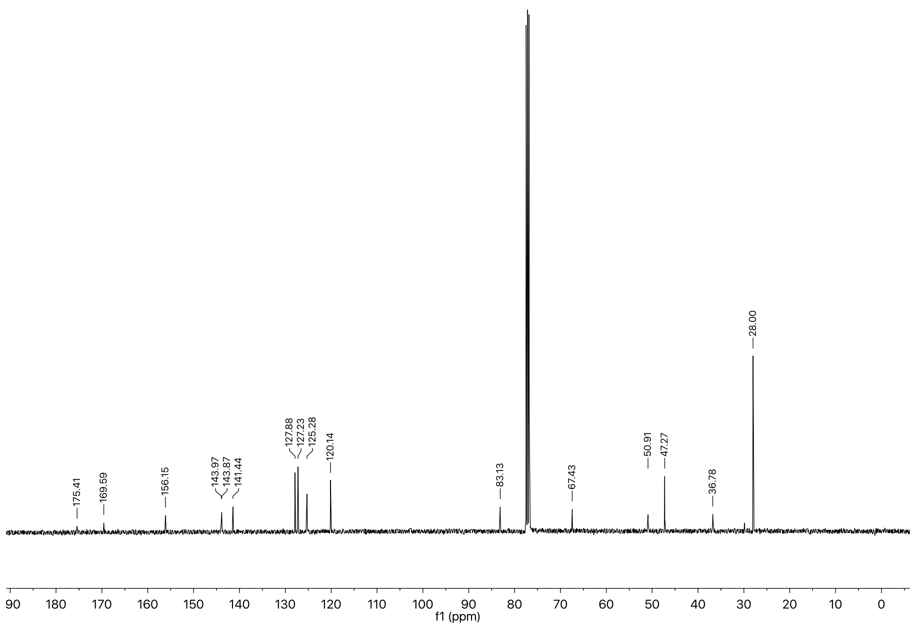

Supplementary Figure 32. **^{1}H and ^{13}C NMR spectra of compound 4.**

N-Fmoc-Asp(CSY)-O*t*Bu 5

^{1}H NMR

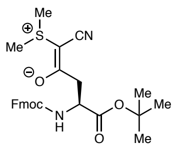

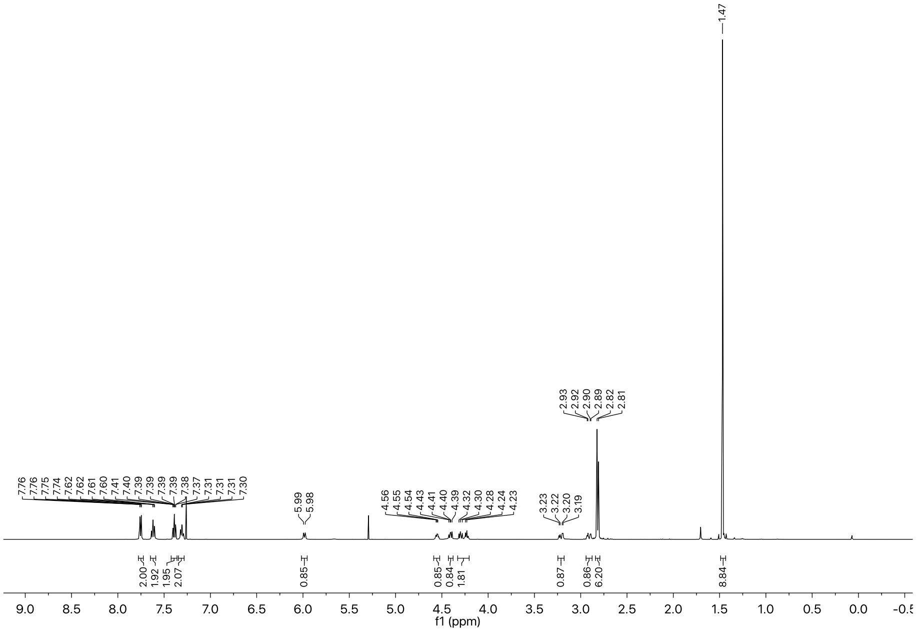

^{13}C NMR

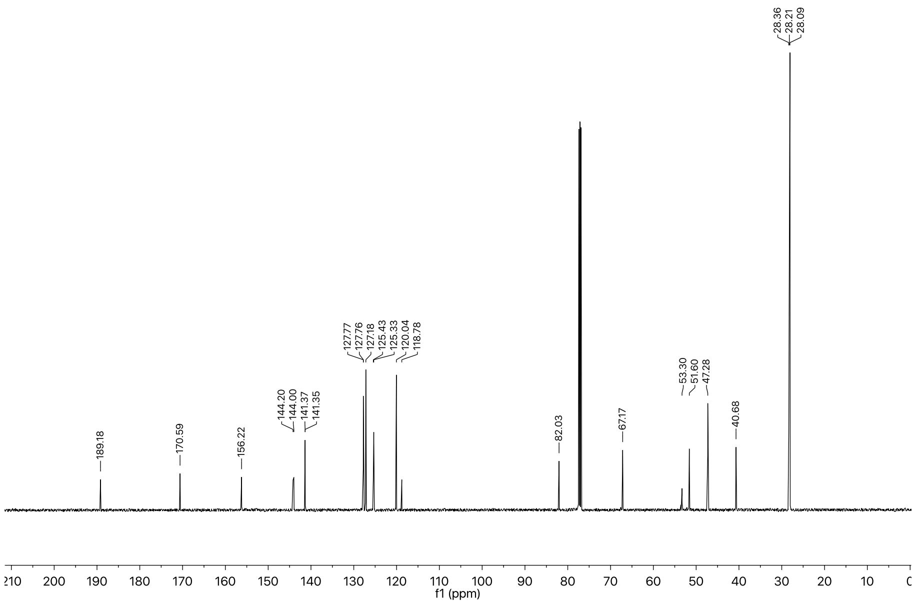

Supplementary Figure 33. **^{1}H and ^{13}C NMR spectra of compound 5.**

4. Supplementary References

1. Tailhades, J. *et al.* Native Chemical Ligation to Minimize Aspartimide Formation during Chemical Synthesis of Small LDLa Protein. *Chem. - A Eur. J.* **22**, 1146–1151 (2016).
2. Lollar, C. T., Krenek, K. M., Bruemmer, K. J. & Lippert, A. R. Ylide mediated carbonyl homologations for the preparation of isatin derivatives. *Org. Biomol. Chem.* **12**, 406–409 (2014).
3. Thuaud, F., Rohrbacher, F., Zwicky, A. & Bode, J. W. Photoprotected Peptide *α* -Ketoacids and Hydroxylamines for Iterative and One-Pot KAHA Ligations: Synthesis of NEDD8. *Helv. Chim. Acta* **99**, 868–894 (2016).
4. Thuaud, F., Rohrbacher, F., Zwicky, A., Bode, J. W., Incorporation of Acid-Labile Masking Groups for the Traceless Synthesis of C-Terminal Peptide α-Ketoacids *Org. Letter* **18**, 3670-3673 (2016).
